# BET proteins are essential for the specification and maintenance of the epiblast lineage in mouse preimplantation embryos

**DOI:** 10.1186/s12915-022-01251-0

**Published:** 2022-03-09

**Authors:** Mami Tsume-Kajioka, Chiharu Kimura-Yoshida, Kyoko Mochida, Yoko Ueda, Isao Matsuo

**Affiliations:** 1 Department of Molecular Embryology, Research Institute, Osaka Women’s and Children’s Hospital, Osaka Prefectural Hospital Organization, 840, Murodo-cho, Izumi, Osaka, 594-1101 Japan; 2 Department of Pediatric and Neonatal-Perinatal Research, Osaka Graduate School of Medicine, Osaka University, Suita, Osaka, 565-0871 Japan

**Keywords:** Mouse, Blastocyst, BET, JQ1, Nanog, Epiblast, Inner cell mass, *Brd4*, *Brd2*, Bromodomain

## Abstract

**Background:**

During mammalian preimplantation development, as the fertilized egg develops and differentiates, three cell lineages become specified: trophectoderm (TE), epiblast, and primitive endoderm (PrE). Through two steps of cell fate decisions, 16-cell blastomeres develop into TE and an inner cell mass (ICM), and thereafter, the latter differentiates into pluripotent epiblast and PrE. Although bromodomain and extra-terminal domain (BET) proteins, such as BRD4, are necessary for the transcriptional activation of genes involved in the maintenance of mouse embryonic stem cells by occupying their enhancers, their roles in the development of mouse preimplantation are unknown.

**Results:**

To evaluate the effect of BET protein deficiency on cell lineage formation, we cultured preimplantation embryos in the presence of JQ1, which blocks the binding of BET bromodomains to acetylated-histones. We found BET inhibition blocked the transcriptional activation of genes, such as *Nanog*, *Otx2*, and *Sox2*, important for the formation of the epiblast lineage in blastocysts. Expression studies with lineage-specific markers in morulae and blastocysts revealed BET proteins were essential for the specification and maintenance of the epiblast lineage but were dispensable for the formation of primarily extraembryonic TE and PrE lineages. Additional Ingenuity Pathway Analysis and expression studies with a transcriptionally active form of signal transducer and activator of the transcription 3 (STAT3) suggested BET-dependent activation was partly associated with the STAT3-dependent pathway to maintain the epiblast lineage. To identify BET proteins involved in the formation of the epiblast lineage, we analyzed mutant embryos deficient in *Brd4*, *Brd2*, and double mutants. Abolishment of NANOG-positive epiblast cells was only evident in *Brd4/Brd2* double-deficient morulae. Thus, the phenotype of JQ1-treated embryos is reproduced not by a *Brd4*- or *Brd2*-single deficiency, but only *Brd4/Brd2*-double deficiency, demonstrating the redundant roles of BRD2 and BRD4 in the specification of the epiblast lineage.

**Conclusions:**

BET proteins are essential to the specification and maintenance of the epiblast lineage by activating lineage-specific core transcription factors during mouse preimplantation development. Among BET proteins, BRD4 plays a central role and BRD2 a complementary role in the specification and maintenance of epiblast lineages. Additionally, BET-dependent maintenance of the epiblast lineage may be partly associated with the STAT3-dependent pathway.

**Supplementary Information:**

The online version contains supplementary material available at 10.1186/s12915-022-01251-0.

## Background

After fertilization, a mouse egg undergoes several rounds of cell division to generate blastocysts that comprised three distinct cell lineages: trophectoderm (TE), epiblast, and primitive endoderm (PrE) [1]. During a 16- to 32-cell morula stage, blastomeres specify the outer TE and inner cell mass (ICM), then from a 32- to 75-cell blastocyst, ICM cells further specify either extraembryonic PrE or pluripotent epiblast lineages; thereafter, epiblast and PrE lineages are maintained and sorted out in the ICM correctly (Additional file 1: Fig. S1A) [1–8]. The formation of the three cell lineages can occur in vitro in a self-organizing manner and is primarily controlled by lineage-specific transcription factors (Additional file 1: Fig. S1A) [1–8]. In terms of the expression of these transcription factors, during 16- to 32-cell stages around E2.75 to E3.25, the blastocyst is differentiating into a CDX2-expressing TE, and OCT3/4-, NANOG-, SOX2-, and GATA6-coexpressing ICM. Then, during the 32- to 75-cell stage around E3.25 to E3.75, ICM cells that co-express NANOG, an epiblast marker, and GATA6, a PrE marker, tend to be expressed in a mutually exclusive manner, analogous to salt and pepper (Additional file 1: Fig. S1A) [4, 8]. Then, after a 75-cell stage around E3.75, ICM cells that mostly express either NANOG or GATA6 are maintained and finally sorted into epiblast and PrE by E4.25, respectively (Additional file 1: Fig. S1A). With respect to the signaling pathways, the first lineage specification into TE and ICM involves Hippo signaling [1–4, 9]. The second lineage specification into epiblast and PrE from the bipotential progenitor ICM involves fibroblast growth factor (FGF) signaling [1–4]. In addition, maintenance of the pluripotent ICM lineage involves signal transducers and activators of the transcription 3 (STAT3) pathway [10]. Thus, such core transcription factors combined with these signaling pathways appear to control the specification and maintenance of these cell lineages primarily in the preimplantation mouse embryo.

The bromodomain and extra-terminal domain (BET) family of proteins, comprising BRD2, BRD3, BRD4, and BRDT, contain tandem bromodomains that can bind acetylated lysine residues of H3 and H4 histones; this functions as an epigenetic reader by regulating gene transcription [11]. Notably, BRD4 recruits the positive transcription elongation factor b (P-TEFb) (i.e., CDK9/Cyclin T complex) and releases paused RNA polymerase II for transcriptional elongation [11–15]. BRD4 is crucial to embryonic stem (ES) cell maintenance [16–19]. In fact, BRD4 activates the transcription of core stem cell genes, such as *Nanog*, *Oct3/4*, and *Prdm14*, by occupying their enhancers in ES cells [17, 18, 20]. Consistent with the roles of *Brd4* in ES cells, *Brd4*-deficient embryos appear to degenerate morphologically just after implantation [21]. Further in vitro culture experiments with *Brd4* small interfering RNA suggest that BRD4 is necessary for proper NANOG protein expression in mouse morulae and blastocysts [17]. These converging lines of evidence suggest that BET proteins, including BRD4, may play important roles in the development of mouse preimplantation.

Here, we show that BET proteins are necessary for the specification and maintenance of the epiblast lineage during the development of mouse preimplantation. Additional Ingenuity Pathway Analysis and comparative studies of genes downregulated by JQ1 and Stattic, inhibitors of pan-BET and STAT3, respectively, suggested such BET-dependent transcriptional activation might be partly associated with the STAT3-dependent pathway to maintain the epiblast lineage. Further genetic studies reveal that among BET proteins, BRD4 plays a central role and BRD2 plays a complementary role to BRD4 in the specification of the epiblast lineage.

## Results

### JQ1 treatment inhibits transcription of specific factors of the epiblast lineage in mouse E3.5 blastocysts

**Fig. 1:**
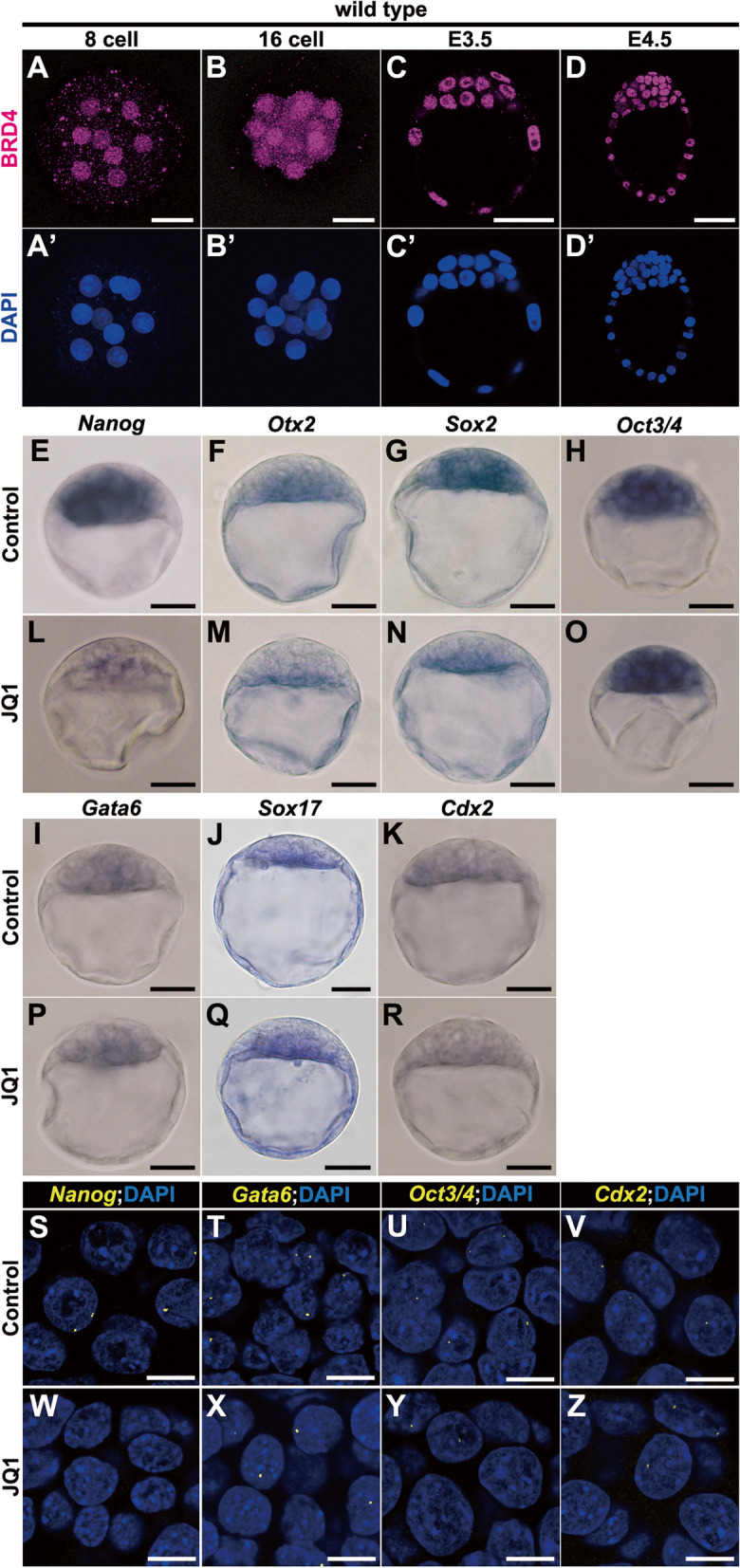
Downregulation of mRNA transcription of epiblast markers in mouse E3.5 blastocysts by JQ1 treatment. **A**–**D**, **A'**–**D'** Immunohistochemical analysis of BRD4 protein (magenta (**A**–**D**)) and DAPI (nuclei, blue (**A’**–**D’**)) staining, in the wild type at 8-cell (**A**, **A’**), 16-cell (**B**, **B’**), E3.5 (**C**, **C’**), and E4.5 (**D**, **D’**) embryos. **E**–**R** Whole-mount in situ hybridization of mouse blastocysts (stage 3) treated without JQ1 (control) (**E**–**K**) or with 5 μM JQ1 (**L**–**R**) for 3 h. Expression of *Nanog* (**E**, **L**), *Otx2* (**F**, **M**), *Sox2* (**G**, **N**), *Oct3/4* (**H**, **O**), *Gata6* (**I**, **P**), *Sox17* (**J**, **Q**), or *Cdx2* (**K**, **R**) mRNAs. **S–Z** RNA-fluorescence in situ hybridization (FISH) of E3.5 blastocysts treated without JQ1 (control; **S**–**V**) or with 5 μM JQ1 (**W**–**Z**) for 1 h. *Nanog*-specific probe (**S**, **W**), *Gata6*-specific probe (**T**, **X**), *Oct3/4*-specific probe (**U**, **Y**), or *Cdx2*-specific probe (**V**, **Z**) (yellow), with DAPI (nuclei, blue) staining. *Nanog* nascent mRNA precursors are absent in the nuclei following treatment with JQ1 (**W**). The sample numbers analyzed for each experiment are indicated in Additional file 19. Scale bars, 10 μm in **S**–**Z**; 25 μm in **A**, **B**, **A’**, **B’**, and **E**–**R**; 40 μm in **C** and **C’**; and 50 μm in **D**, **D’**

To investigate if BET family proteins are involved in the development of mouse preimplantation, we first analyzed BRD4 protein expression using specific antibodies, because BRD4 has been suggested to be necessary for early mouse embryogenesis [17, 21]. We found that BRD4 was ubiquitously expressed in the nuclei throughout preimplantation stages (Fig. 1A–D, A’–D’). At the 8-cell stage prior to the first lineage specification, BRD4 had already accumulated in the nuclei of all blastomeres (Fig. 1A, A’). Thereafter, it continued to be localized in the nuclei of all cell lineages, from 16-cell to E4.5 (Fig. 1B–D, B’–D’).

Then, to evaluate if BET family proteins play a role in the formation of the cell lineage comprehensively and in a timely manner, we exploited (+)-JQ1 (JQ1), which competitively associates with the bromodomains of BET proteins; in particular, it shows the highest affinity with the first bromodomain of BRD4 and blocks binding to acetylated histones [22]. JQ1 can immediately and reversibly block bromodomain function allowing us to analyze BET function in real time in preimplantation embryos. We collected wild-type blastocysts at E3.5 and cultured these embryos in vitro in the presence or absence of JQ1 (Additional file 1: Fig. S1B). Since BET proteins, including BRD4, have been shown to regulate transcription [12–14, 23], we cultured these blastocysts for 3 h to detect early effects on transcription and then examined several lineage-specific markers, at the mRNA level, in JQ1-treated blastocysts (Fig. 1E–R). The expression of *Nanog* transcripts, an epiblast marker, was severely reduced with JQ1 treatment (Fig. 1E, L). Similarly, transcripts of *Otx2* and *Sox2*, which are epiblast and ICM markers, respectively [3, 24–26], were also reduced in JQ1-treated blastocysts (Fig. 1F, G, M, N). However, *Oct3/4* mRNA, a marker for an undifferentiated state; two PrE markers (*Gata6* and *Sox17*); and a TE marker (*Cdx2*) were not reduced in JQ1-treated blastocysts (Fig. 1H–K, O–R). These findings indicated that JQ1 treatment decreased gene expression in the epiblast lineage, but not in extraembryonic TE and PrE lineages, in mouse blastocysts; however, *Oct3/4* expression was not affected by JQ1 treatment.

To validate whether such a reduction of mRNA expression is primarily due to a failure of transcription, we analyzed the transcription of mRNA precursors (i.e., nascent RNAs of genes) in genomic loci by means of RNA fluorescence in situ hybridization after JQ1 treatment for 1 h to detect an immediate effect on transcription (Fig. 1S–Z). In control E3.5 blastocysts, nascent *Nanog* mRNA precursors were detectable at *Nanog* loci within the nuclei of cells of the ICM (Fig. 1S). However, *Nanog* mRNA precursors were not evident in the nuclei of JQ1-treated blastocysts (Fig. 1W). Conversely, nascent RNA precursors of *Gata6*, *Oct3/4*, and *Cdx2* genes, which were not reduced at the mRNA level, were detectable at their respective genomic loci within the nuclei of JQ1-treated blastocysts at a comparable level to that of control blastocysts (Fig. 1T–V, X–Z). These findings are comparable to the proposed transcriptional function of BET proteins in that JQ1 treatment inhibits transcriptional activation of BET-target genes.

### JQ1 treatment blocks specification and maintenance of the epiblast lineage

**Fig. 2:**
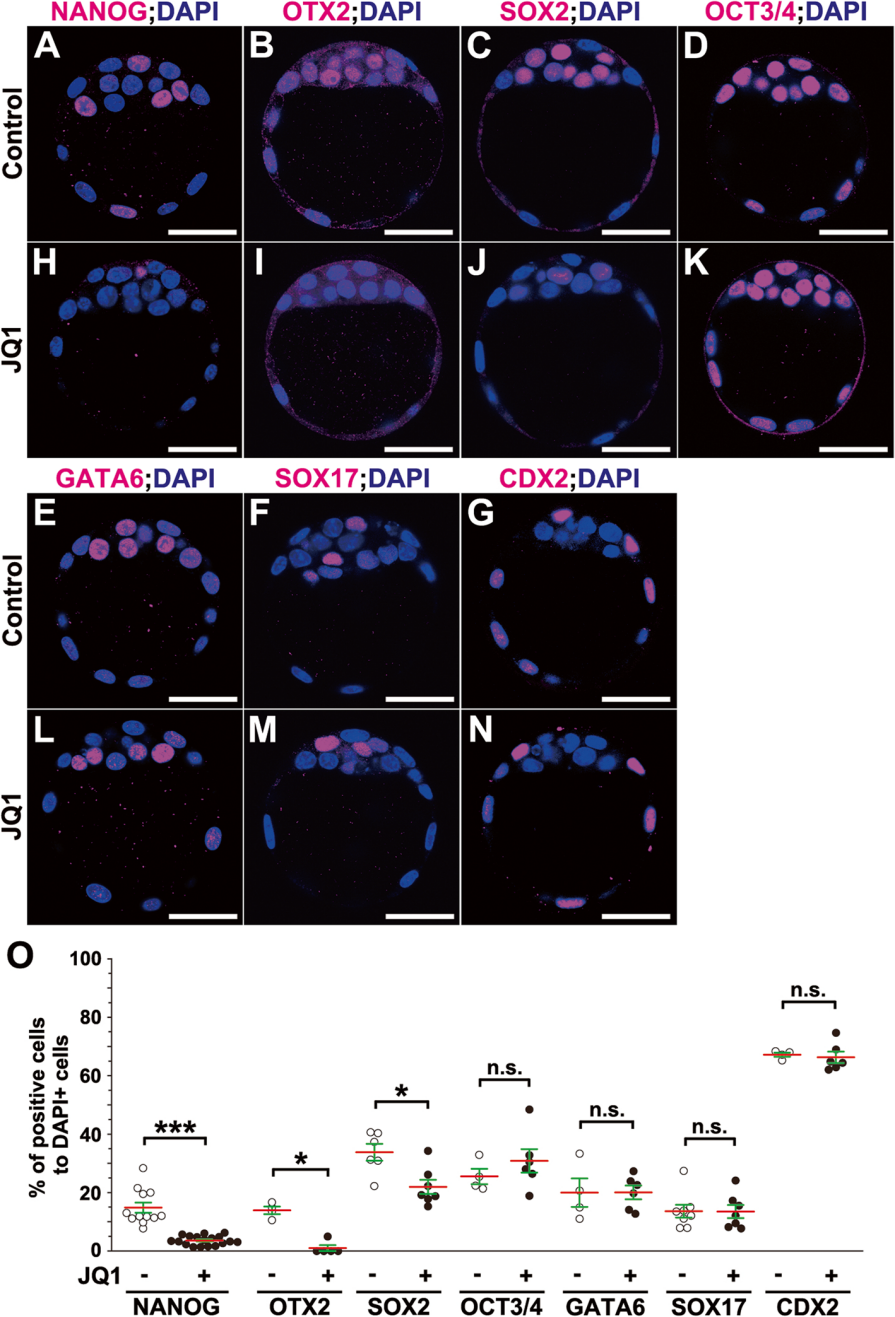
Downregulation of epiblast-lineage markers in mouse blastocysts by JQ1 treatment (stages 3 to 4). **A–N** Immunohistochemical analysis of mouse blastocysts (stage 3) treated without JQ1 (control) (**A**–**G**) or with 5 μM JQ1 (**H**–**N**) for 6 h. NANOG (**A**, **H**), OTX2 (**B**, **I**), SOX2 (**C**, **J**), OCT3/4 (**D**, **K**), GATA6 (**E**, **L**), SOX17 (**F**, **M**), or CDX2 (**G**, **N**) (magenta), with DAPI (nuclei, blue) staining. **O** The ratio of numbers of NANOG-, OTX2-, SOX2-, OCT3/4-, GATA6-, SOX17-, or CDX2-expressing cells to DAPI-positive cells (nuclei) (two-tailed Mann-Whitney’s *U*-test; ***p < 0.001, *p < 0.05; n.s., not significant; OCT3/4, *p* = 0.476; GATA6, *p* = 0.914; SOX17, *p* = 0.954; CDX2, *p* = 0.476). Red lines indicate the mean values, and green lines represent the SE bars. Individual values of marker-expressing cells are provided in Additional file 15. The sample numbers analyzed for each experiment are indicated in Additional file 19. Scale bars, 40 μm in **A**–**N**

Next, to more precisely investigate what cell fates are affected with JQ1 treatment, we analyzed the expression of lineage-specific markers in a single-cell resolution at the protein level (Fig. 2). In this study, to clarify the role of BET proteins in the specification and maintenance of a cell lineage, we named the four preimplantation embryo stages, i.e., from 8 to 128 cells was “stage 1” to “stage 4” based on the developmental character of NANOG-positive cells [1–8] (Additional file 1: Fig. S1A). In “stage 1” (8 to 16 cells), NANOG, GATA6, and CDX2 were co-expressed in totipotent blastomere cells. In “stage 2” (16 to 32 cells), totipotent blastomeres were specified to ICM and TE cells in morulae. Three types of cells were found: blastomere cells co-expressing NANOG, GATA6, and CDX2 were decreased, but those either expressing or not expressing CDX2 progressively increased over time. In “stage 3” (32 to 75 cells), ICM cells were specified to epiblast and PrE cells in blastocysts. Three types of cells were found: ICM cells co-expressing NANOG and GATA6 were decreased, but ICM cells that expressed either NANOG or GATA6 (i.e., in a mutually exclusive, salt and pepper-like manner) progressively and gradually increased over time [8] (Additional file 1: Fig. S1C–C”, D). In “stage 4” (75 to 128 cells), ICM-derived cells expressing either NANOG or GATA6 were mostly present, and epiblasts and PrE cells were maintained and sorted out within the ICM of blastocysts.

We then collected wild-type blastocysts at E3.5 that consisted of 45 to 64 cells (stage 3), when ICM specifies into epiblast and PrE lineages, and cultured these blastocysts for 6 h (from stage 3 to partly stage 4 in Additional file 1: Fig. S1A–D). After in vitro culture, NANOG protein was detectable in cells of some portions of the ICM in control blastocysts (Fig. 2A). Consistent with the above mRNA expression, the protein expression of NANOG was greatly diminished in JQ1-treated blastocysts compared to that of control blastocysts (Fig. 2H). To further estimate the effect of JQ1 treatment on cell fate more quantitatively, we counted cell numbers expressing these specific markers. Notably, the total number of cells, as determined by 4′,6-diamidino-2-phenylindole (DAPI)-positive cells, remained unchanged between JQ1-treated and JQ1-untreated blastocysts (Additional file 1: Fig. S1D). As expected, the ratio of the number of NANOG-positive epiblast lineage cells to DAPI-positive cells in blastocysts was sharply decreased with JQ1 treatment (Fig. 2O). As OTX2 expression appeared to be found in some ICM cells corresponding to NANOG-positive cells as reported previously (Fig. 2B) [26], most OTX2 protein expression was completely lost in JQ1-treated blastocysts, similar to NANOG expression (Fig. 2I). Additionally, the ratio of OTX2-positive epiblast lineage cells to DAPI-positive cells in blastocysts was also diminished with JQ1 treatment (Fig. 2O). SOX2 expression was found in all ICM cells of control blastocysts (Fig. 2C) [25] while SOX2 protein expression was reduced with JQ1 treatment to some extent (Fig. 2J). Accordingly, the ratio of SOX2-positive cells to DAPI-positive cells was also reduced with JQ1 treatment (Fig. 2O). Conversely, OCT3/4 protein expression was found in all ICM cells while it was not decreased in JQ1-treated blastocysts (Fig. 2D, K). Moreover, the expression of GATA6 and SOX17, two PrE markers, and CDX2, a TE marker, appeared to be unchanged (Fig. 2E–G, L–N). Since reciprocal interaction between NANOG and GATA6 expression has been reported in stage 3 [27, 28], we tested whether the ratio of GATA6-positive PrE lineage cells to DAPI-positive cells was increased or not. No significant difference was found in the ratio of GATA6-positive cells to DAPI-positive cells between JQ1-treated and control blastocysts (Fig. 2O). Similarly, the ratios of OCT3/4-, SOX17-, and CDX2-positive cells to DAPI-positive cells were neither decreased nor increased with JQ1 treatment (Fig. 2O). These findings suggest that JQ1 treatment primarily blocks specification and maintenance of the epiblast lineage (stages 3 and 4), rather than that of PrE or TE, two extraembryonic lineages.

### JQ1 treatment blocks earlier specification of ICM and epiblast lineages reversibly

**Fig. 3:**
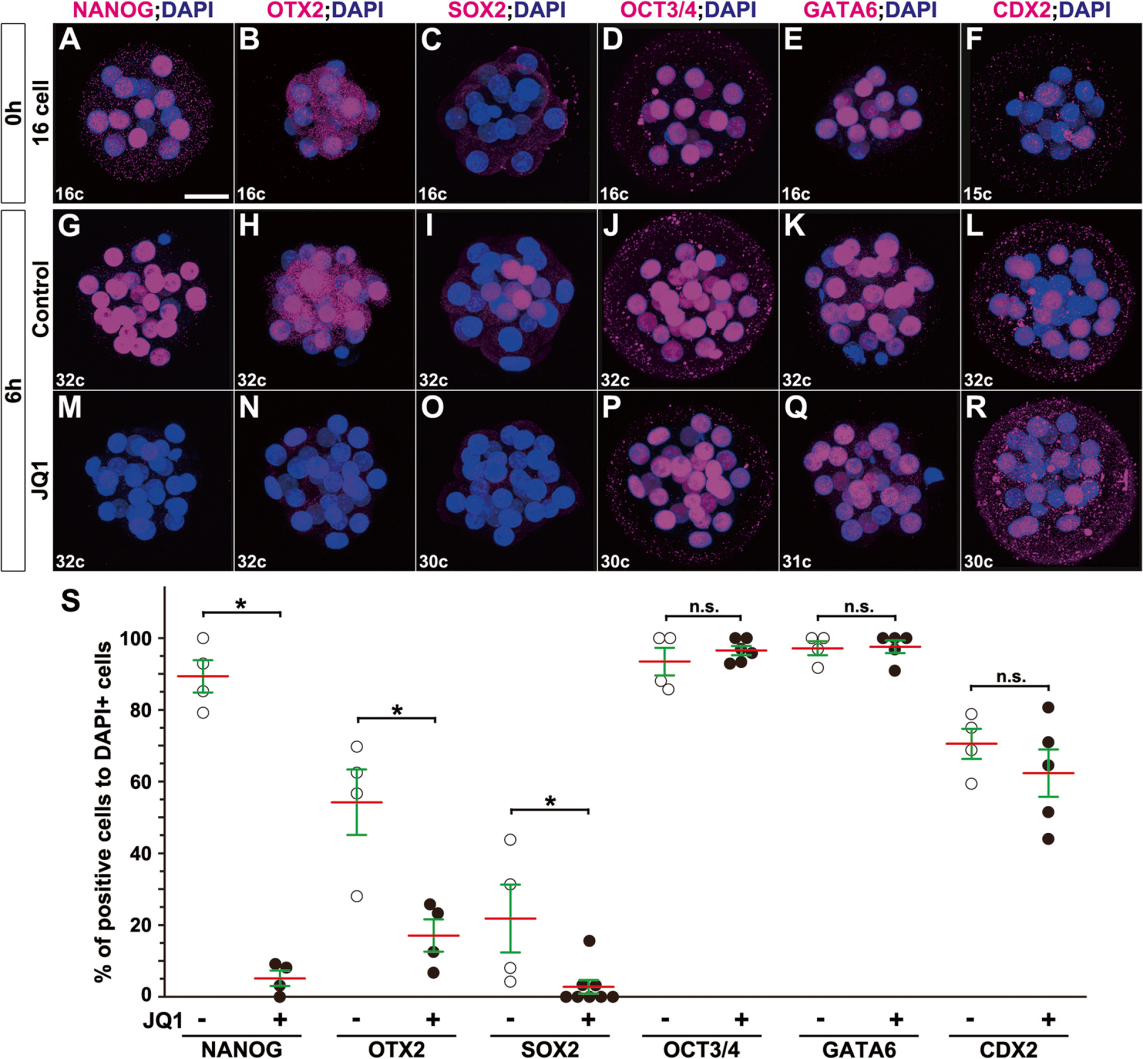
Downregulation of epiblast-lineage markers in mouse morulae by JQ1 treatment (stage 2). **A–R** Immunohistochemical analysis of mouse 16-cell morulae before treatment (**A**–**F**), treated without JQ1 (control) (**G**–**L**) or with 5 μM JQ1 (**M**–**R**) for 6 h. NANOG (**A**, **G**, **M**), OTX2 (**B**, **H**, **N**), SOX2 (**C**, **I**, **O**), OCT3/4 (**D**, **J**, **P**), GATA6 (**E**, **K**, **Q**), or CDX2 (**F**, **L**, **R**) (magenta), with DAPI (nuclei, blue) staining. The bottom left numerical character of each panel denotes the number of DAPI-positive cells in the embryos shown. **S** The ratio of numbers of NANOG-, OTX2-, SOX2-, OCT3/4-, GATA6-, or CDX2-expressing cells to DAPI-positive cells (nuclei) (two-tailed Mann-Whitney’s *U*-test; *p < 0.05; n.s., not significant; OCT3/4, *p* = 0.660; GATA6, *p* = 1.000; CDX2, *p* = 0.556). Red lines indicate the mean values, and green lines represent the standard error (SE) bars. The numbers of marker-expressing cells are shown in Additional file 15. The sample numbers analyzed for each experiment are shown in Additional file 19. Scale bars: 25 μm in **A**–**R**

Since the epiblast-specific lineage markers, including NANOG, that are already expressed in earlier stages and maintained, we next validated if JQ1 could affect the much earlier specification of ICM and TE lineages (stage 2). We subsequently collected around 16-cell early morulae, cultured them with JQ1 for 6 h, and analyzed lineage-specific markers in late morulae (from stage 2 to stage 2; Fig. 3). The protein expression of NANOG was evident in most blastomeres at the 16-cell morula stage before treatment (Fig. 3A). However, NANOG expression was greatly diminished in JQ1-treated morulae compared to that of controls (Fig. 3G, M). Additionally, OTX2 protein expression was observed in half of the blastomeres in control morulae while expression was almost abolished in JQ1-treated morulae similar to NANOG expression (Fig. 3B, H, N). Notably, SOX2 expression appeared to be normally induced in some portions of blastomeres in 32-cell morulae but not in 16-cell morulae (Fig. 3C, I). However, SOX2 protein expression was not induced in JQ1-treated morulae (Fig. 3O). Similar to that observed in blastocysts, OCT3/4, GATA6, and CDX2 protein expression were unaffected in JQ1-treated morulae (Fig. 3D–F, J–L, P–R). Consistently, the ratios of NANOG-, OTX2-, and SOX2-positive cells to DAPI-positive cells were diminished quantitatively while those of OCT3/4, GATA6, and CDX2 were unchanged after JQ1 treatment (Fig. 3S). These findings indicate that JQ1 treatment can also block the specification of the ICM lineage for the epiblast formation (stage 2).

NANOG protein is normally expressed in all blastomeres as early as at the 8-cell stage (stage 1) [29]. Therefore, to further explore the effects of JQ1 treatment, we treated 8-cell embryos for 6 h with JQ1 and similarly analyzed 16-cell morulae (from stage 1 to stage 1) using lineage-specific markers. Consequently, we found that NANOG expression was severely diminished with JQ1 treatment while GATA6 and OCT3/4 expression was unchanged in JQ1-treated morulae (Additional file 2: Fig. S2A–F). Accordingly, the ratio of the number of NANOG-positive cells to DAPI-positive cells was completely diminished quantitatively, while those of OCT3/4 and GATA6 were not, after JQ1 treatment (Additional file 2: Fig. S2G). These findings support the notion that JQ1 treatment can affect the earlier specification of the ICM lineage from the beginning.

**Fig. 4:**
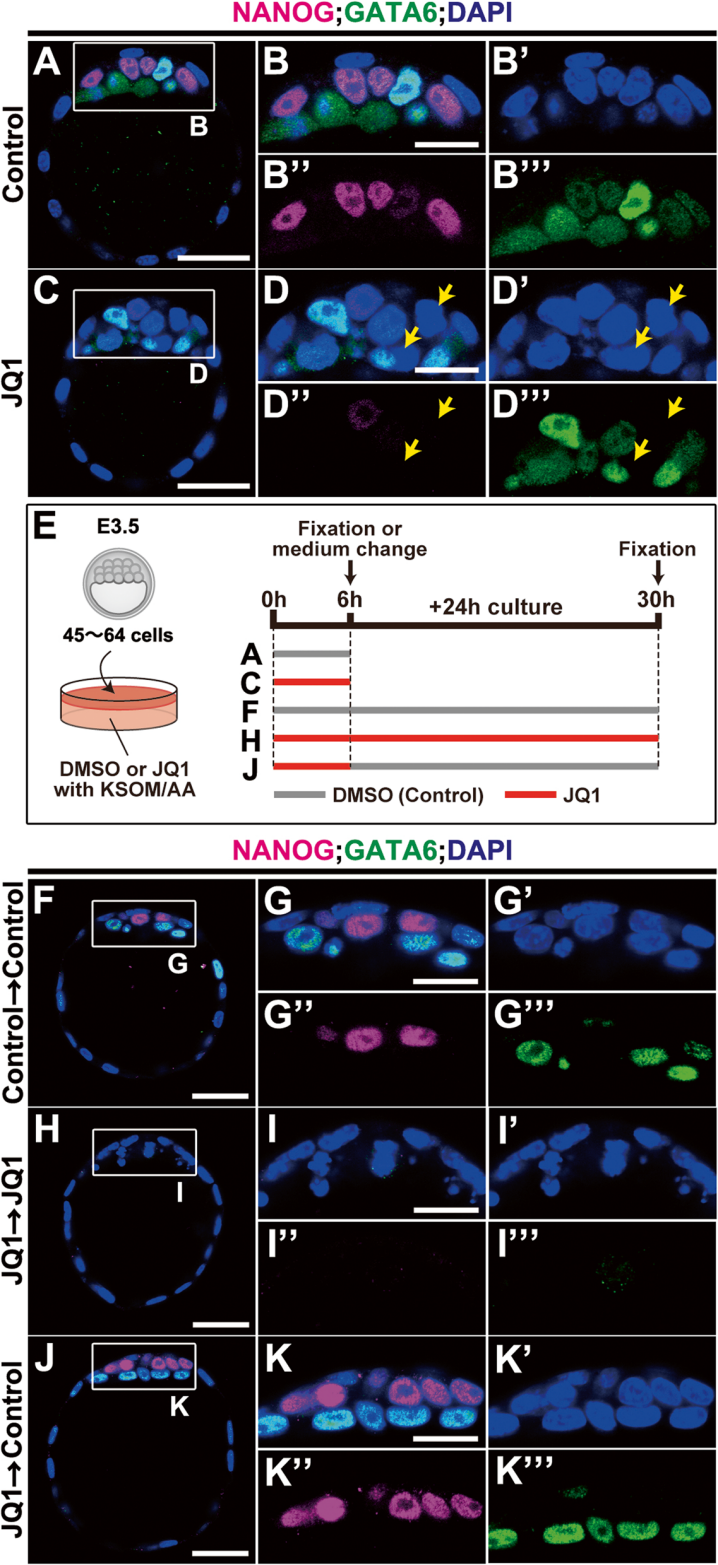
NANOG- and GATA6-double-negative cells are emerged from JQ1-treated blastocysts and appear to re-induce NANOG-positive cells reversibly (stages 3 to 4). **A–D**, **B’**, **B”**, **B”’**, **D’**, **D”**, **D”’** NANOG- and GATA6-doube-negative cells emerged after JQ1 treatment (yellow arrows). Immunohistochemical analysis of NANOG (magenta) and GATA6 (green), with DAPI (nuclei, blue) staining in control (**A**, **B**, **B’**, **B”**, **B”’**) and 5 μM JQ1-treated blastocysts (**C**, **D**, **D’**, **D”**, **D”’**) for 6 h at E3.5. **E** Schematic illustration of the experimental strategy, with or without JQ1, for an additional 24-h culture. **F**–**K**, **G’**, **G”**, **G”’**, **I’**, **I”**, **I”’**, **K’**, **K”**, **K”’** NANOG- and GATA6-double-negative cells are re-specified into NANOG-positive cells after JQ1 removal. Immunohistochemical analysis of NANOG (magenta) and GATA6 (green), with DAPI (blue) staining, in mouse blastocysts at E3.5 treated without JQ1 for 30 h (**F**, **G**, **G’**, **G”**, **G”’**), with 5 μM JQ1 for 30 h (**H**, **I**, **I’**, **I”**, **I”’**), and with 5 μM JQ1 for 6 h and without JQ1 for 24 h (**J**, **K**, **K’**, **K”**, **K”’**). The sample numbers analyzed for each experiment are indicated in Additional file 19. Scale bars, 20 μm in **B**, **B’**, **B”**, **B”’**, **D**, **D’**, **D”**, **D”’**, **G**, **G’**, **G”**, **G”’**, **I**, **I’**, **I”**, **I”’**, **K**, **K’**, **K”**, **K”’**; 40 μm in **A**, **C**, **F**, **H**, and **J**. DMSO, dimethyl sulfoxide

The following three lines of evidence: a reduced number of NANOG-positive cells and an unaltered number of total DAPI-positive and GATA6-positive cells by JQ1 treatment, suggest the presence of both NANOG- and GATA6-double-negative cells in JQ1-treated blastocysts (Fig. 2O, Additional file 1: Fig. S1D). Consistent with this assumption, such double-negative cells were clearly observed in JQ1-treated blastocysts (stages 3 to 4) (Fig. 4A–D, B’, B”, B”’, D’, D”, D”’). To further test if these JQ1-treated NANOG-negative cells still retained the competence of the epiblast lineage, we further cultured these JQ1-treated blastocysts for 24 h in the absence of JQ1 and examined the expression of NANOG and GATA6 (stage 4; Fig. 4E). Control ICM cells were able to be specified into NANOG-positive and GATA6-positive cells after a 30-h culture without JQ1 treatment normally (Fig. 4F, G, G’, G”, G”’). In contrast, JQ1 treatment for 30 h appeared to repress both NANOG and GATA6 expression (Fig. 4H, I, I’, I”, I”’). This failure in GATA6 expression might be due to the necessity of a reciprocal interaction between epiblast and PrE lineages [1–4, 8]. However, an additional 24-h culture without JQ1, after an initial 6 h of JQ1 treatment, led to the re-induction of NANOG expression in ICM cells (Fig. 4J, K, K’, K”, K”’). These findings suggest that the inhibitory effect of JQ1 on specification and maintenance of the epiblast lineage is reversible as long as it is within 6 h and ICM cells can still retain competency and recover to re-specify the epiblast lineage once again, even after JQ1 treatment.

### BET proteins activate transcription of genes partly associated with the STAT3-dependent pathway in the epiblast lineage

**Fig. 5:**
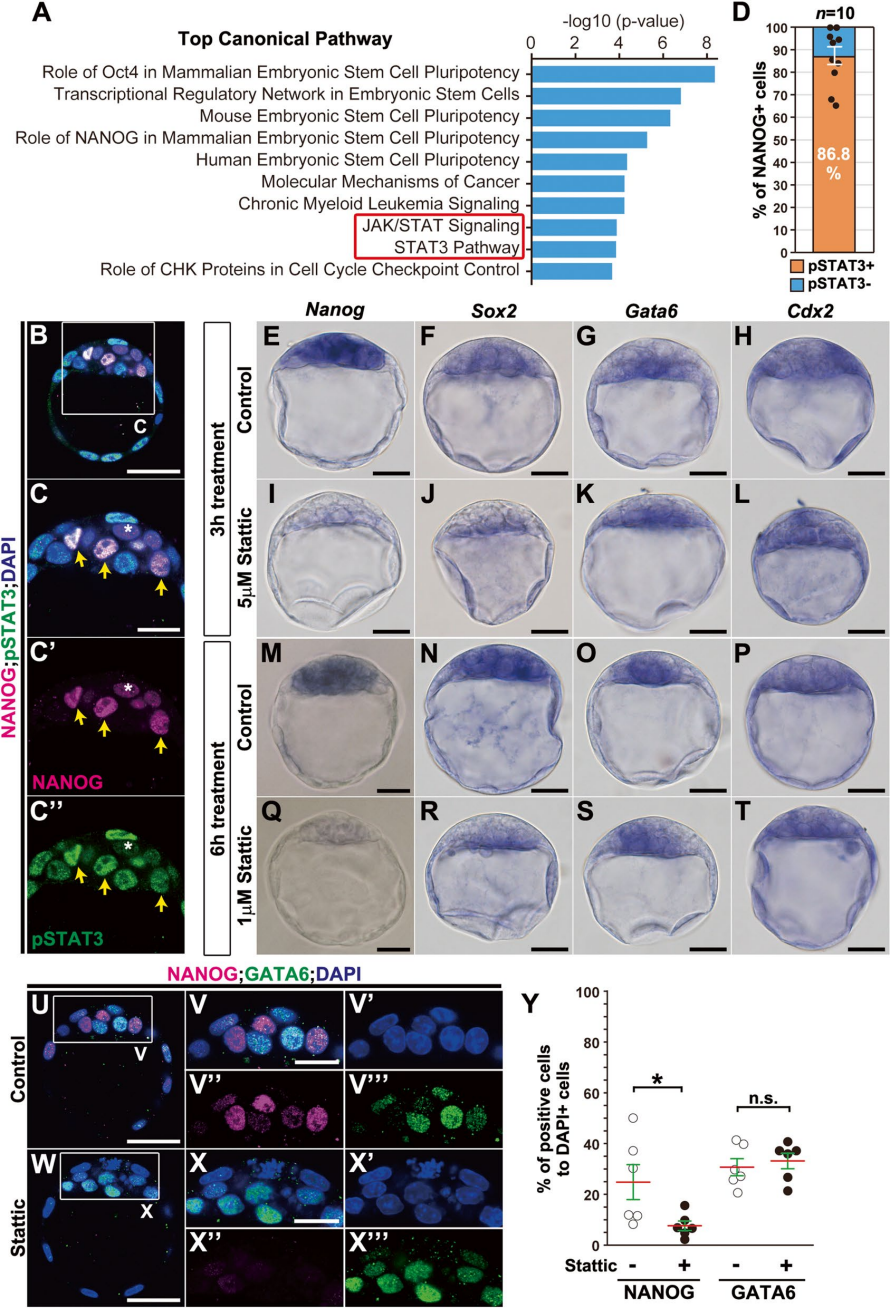
Downregulation of epiblast-lineage markers by JQ1 treatment associated with STAT3 inhibition (stages 3 to 4). **A** Representation of the top ten canonical pathways by Ingenuity Pathway Analysis (IPA). **B**, **C**, **C’**, **C”** Immunohistochemical analysis of NANOG (magenta) and pSTAT3 (green), with DAPI (blue) staining, in mouse blastocysts at E3.5. NANOG-positive cells that express pSTAT3 (yellow arrows) but do not express (asterisks) in the nuclei. **D** The ratio of pSTAT3-expressing and non-expressing cells to all NANOG-positive cells. White lines represent the SE bars. *n* indicates the number of blastocysts analyzed. **E**–**T** Whole-mount in situ hybridization of mouse blastocysts treated without Stattic (**E**–**H**, **M**–**P**) or with 5 μM Stattic (**I**–**L**) for 3 h or 1 μM Stattic (**Q**–**T**) for 6 h (stages 3 to 4). Expression of *Nanog* (**E**, **I**, **M**, **Q**), *Sox2* (**F**, **J**, **N**, **R**), *Gata6* (**G**, **K**, **O**, **S**), or *Cdx2* (**H**, **L**, **P**, **T**) mRNAs. **U**–**X**, **V’**, **V”**, **V”’**, **X’**, **X”**, **X”’** Immunohistochemical analysis of NANOG (magenta) and GATA6 (green), with DAPI (nuclei, blue) staining of mouse blastocysts treated without Stattic (**U**, **V**, **V’**, **V”**, **V”’**) or with 1 μM Stattic (**W**, **X**, **X’**, **X”**, **X”’**) for 6 h (stages 3 to 4). **Y** The ratios of the numbers of NANOG- or GATA6-expressing cells to DAPI-positive total cells (nuclei) treated without Stattic or with 1 μM Stattic for 6 h of E3.5 blastocysts (two-tailed Mann-Whitney’s *U*-test; *p < 0.05; n.s., not significant; *p* = 0.748). Red lines indicate the mean values, and green lines represent the SE bars. Individual values of marker-expressing cells are provided in Additional file 15. The sample numbers analyzed for each experiment are indicated in Additional file 19. Scale bars, 20 μm in **C**, **C’**, **C”**, **V**, **V’**, **V”**, **V”’**, **X**, **X’**, **X”**, **X”’**; 25 μm in **E**–**T**; 40 μm in **B**, **U**, **W**

Given that severe abnormalities in epiblast formation were evident, including specification and maintenance of the epiblast lineage, but neither extraembryonic TE nor PrE lineages after JQ1 treatment (Figs. 1, 2, 3, and 4), we hypothesized that the genes activated by BET proteins might mediate specific signaling pathways involving the formation of the epiblast lineage. To identify the biological pathways associated with genes downregulated by JQ1 treatment, we performed DNA microarrays, with control and JQ1 treatments of E3.5 blastocysts cultured for 3 h, to detect direct targets of BET proteins (stage 3 to stage 3) and compared RNA expression profiles between the two samples (Additional file 3: Fig. S3A). We found hierarchical clustering clearly distinguished between control and JQ1-treated samples (Additional file 3: Fig. S3B), meaning that JQ1 treatment led to the alteration of transcription profiles, i.e., 288 genes were downregulated, and 113 genes were upregulated by a greater than twofold change compared to control RNA (Additional file 3: Fig. S3B; Additional file 4). To verify the expression of genes that were significantly downregulated in the microarray (Additional file 3: Fig. S3C), we examined four markedly downregulated genes, *Arid5b*, *Pramel6*, *Pramel7*, and *Trim43a*. Consistently, these genes appeared to be expressed in control blastocysts (Additional file 3: Fig. S3D–G) while their expression was diminished in JQ1-treated blastocysts (Additional file 3: Fig. S3H–K). Collectively, these findings demonstrate that JQ1 treatment downregulates a considerable number of genes, primarily in the mouse blastocyst. Subsequently, we analyzed differentially expressed gene profiles with microarray using Ingenuity Pathway Analysis (IPA) and extracted the top ten dysregulated canonical pathways (Fig. 5A). We found that the top five specific pathways implicated by downregulated genes related to ES cell pluripotency (Fig. 5A). The highest associated pathway “role of Oct4 in mammalian embryonic stem cell pluripotency” may be consistent with the finding that BRD4 activates OCT4-target genes for pluripotency in ES cells ([30]; see the “Discussion” section). Among the top 10 pathways, we homed in on the Janus kinase (JAK)/STAT and STAT3 pathways since STAT3 is reported to be necessary to maintain *Nanog* expression [10].

To estimate the activity of STAT3 associated with the specification and maintenance of the epiblast lineage, we analyzed the expression of a phosphorylated form of STAT3 (tyrosine 705; pSTAT3), a crucial marker for a transcriptionally active form of STAT3 [31], together with NANOG expression in blastocysts (stage 3) in detail. Consistently, we found that nuclear expression of pSTAT3 was heterogeneously expressed while 86.8% of NANOG-positive cells expressed nuclear pSTAT3 in ICM cells (Fig. 5B–D, C’, C’’). This result suggests that pSTAT3 expression in the nuclei may be necessary for NANOG expression.

To evaluate whether the genes downregulated by JQ1 treatment are involved in STAT3 function, we exploited Stattic, an inhibitor of STAT3 [32], and explored the effective conditions necessary for Stattic to inhibit pSTAT3 expression in ICM (Additional file 5: Fig. S4). Given that its IC50 was 5.1 μM in a cell-free assay [32] and our in vitro culture durations used for expression analysis were mainly 3 and 6 h, we tested the pSTAT3 expression treated with different Stattic concentrations after 3- or 6-h cultures (Additional file 5: Fig. S4A–G). We found that 5 μM Stattic treatment for either 3 or 6 h reduced pSTAT3 expression while 1 μM Stattic treatment for only 6 h reduced pSTAT3 (Additional file 5: Fig. S4A–G). However, a 6-h culture with 5 μM Stattic treatment appeared to induce severe defects in embryo morphology with developmental retardation while 1 μM Stattic treatment for 6 h and 5 μM Stattic treatment for 3 h did not appear to lead to morphological abnormalities (Additional file 5: Fig. S4D’, E’, F’). Thus, we compared the expression of the lineage-specific markers, *Nanog*, *Sox2*, *Gata6*, and *Cdx2*, in Stattic-treated blastocysts at the mRNA level under two conditions (stages 3 to 4). We found that *Nanog* and *Sox2* transcripts were reduced with 5 μM Stattic treatment in a 3-h culture (Fig. 5E, F, I, J). However, both *Gata6* and *Cdx2* transcripts were not reduced under this condition (Fig. 5G, H, K, L). Similarly, 1 μM Stattic treatment for 6 h yielded similar expression profiles: *Nanog* and *Sox2* transcripts were diminished while *Gata6* and *Cdx2* transcripts were not (Fig. 5M–T). These findings suggest that 1 μM Stattic treatment for 6 h can effectively inhibit the STAT3-dependent pathway similar to 5 μM Stattic treatment for 3 h.

To further evaluate if the specification of the epiblast lineage is affected as observed in JQ1-treated embryos, we analyzed the protein expression of epiblast and PrE lineage-specific markers in Stattic-treated blastocysts with 1 μM Stattic treatment for 6 h (stages 3 to 4). Consequently, we found that NANOG, but not GATA6, expression was reduced in the ICM (Fig. 5U–X, V’, V”, V”’, X’, X”, X”’). More quantitatively, the ratio of the number of NANOG-positive cells to DAPI-positive cells, but not GATA6-positive cells, was decreased in Stattic-treated blastocysts (Fig. 5Y). These findings suggest treatment with 1 μM Stattic for 6 h can block specification and maintenance of the epiblast lineage similar to JQ1 treatment.

To further validate if JAK signaling can also contribute to the formation of the epiblast lineage, we exploited TG101209, an inhibitor of JAK2, and analyzed NANOG and GATA6 expression in blastocysts (stages 3 to 4). Consistently, TG101209 treatment was able to effectively downregulate pSTAT3 expression in ICM (Additional file 5: Fig. S4H–I’). Notably, NANOG, but not GATA6, expression was reduced, and the ratio of the number of NANOG-positive cells to DAPI-positive cells was diminished (Additional file 5: Fig. S4J–N). These findings, together, suggest that the formation of the NANOG-positive epiblast lineage, but not the GATA6-positive PrE lineage, can be affected by blocking STAT3- and JAK2-mediated signaling; likewise, with JQ1 treatment. These aforementioned results suggest that JAK2/STAT3 inhibition may affect the specification and maintenance of the epiblast lineage (stages 3 and 4).

To validate if JQ1 treatment can downregulate the activity of STAT3, we examined pSTAT3 expression in JQ1-treated blastocysts and found pSTAT3 was markedly reduced in the entire ICM with JQ1 treatment (Additional file 6: Fig. S5A–B’; stages 3 to 4). To verify if a reduction in pSTAT3 occurred at the post-translational level, we re-assessed the expression level of *Stat3* transcripts by microarray and found that these were neither down- nor upregulated significantly between control and JQ1-treated blastocysts (fold change, − 1.04, *p* = 0.935; Additional file 4). In fact, the expression of pan-STAT3 was apparently unchanged among control, JQ1-treated, and Stattic-treated blastocysts (Additional file 6: Fig. S5E–G’). Thus, phosphorylation of STAT3 might be blocked by inhibitor treatment. These findings, together, further suggest that BET that is involved in the formation of the epiblast lineage is associated with STAT3 activation.

To further address how BET proteins can promote phosphorylation of nuclear STAT3, we exploited excess leukemia inhibitory factor (LIF), one of the chemical activators of the STAT3 pathway. Since a possible interaction between STAT3 and BET proteins may be those BET proteins that are necessary to express ligands such as LIF for STAT3 activation, we tested whether excess LIF could restore STAT3 activity in the presence of JQ1. In fact, *Lif* mRNA expression was found to be reduced in JQ1-treated blastocysts (stage 3 to stage 3; Additional file 4 and see below). We then cultured blastocysts (stages 3 to 4) in the presence of excess LIF and found that pSTAT3 was upregulated in all ICM cells as compared with the control (Additional file 6: Fig. S5H, H’, I, I’). Coincidentally, *Nanog* transcripts, which were decreased by JQ1 and Stattic treatment, appeared to be increased (Additional file 6: Fig. S5K, L). Thereafter, we cultured blastocysts in combination with JQ1 and excess LIF. However, neither phosphorylation of STAT3 nor *Nanog* expression were restored and, in fact, were almost lost in the ICM, even in the presence of excess LIF (Additional file 6: Fig. S5J, J’, M). These findings indicate that excess LIF is not sufficient to rescue or complement the expression of BET-target genes. Collectively, these findings suggest that BET proteins might activate STAT3-associated gene transcription downstream of the LIF receptor and upstream of STAT3 in the nucleus or via a LIF-independent non-canonical JAK/STAT pathway [33, 34].

### JQ1-sensitive genes have partly overlapped with transcriptional activation of Stattic-sensitive genes for specification and maintenance of epiblast lineage

Although the aforementioned findings suggest the association between BET-target genes and the Stattic-dependent pathway, we found some of BET-target genes such as *Arid5b* and *Trim43a* transcripts were not downregulated by Stattic-treatment (Additional file 3: Fig. S3D, G, H, K; Additional file 7: Fig. S6A–H). Then, to more comprehensively elucidate downstream target genes transcriptionally activated by BET proteins in the STAT3-dependent pathway in blastocysts, we cultured E3.5 blastocysts with JQ1 or 1 μM Stattic for 6 h (from stage 3 to partly stage 4) and performed RNA sequencing (RNA-seq) analysis (Additional file 7: Fig. S6I, Additional files 8 and 9). Consequently, we identified 1373 genes that were downregulated by JQ1 treatment (11.5% of all expressed genes) and 312 genes that were downregulated by Stattic treatment (2.6% of all expressed genes; Additional file 7: Fig. S6J), defined as JQ1-sensitive and Stattic-sensitive genes, respectively. Importantly, we found 110 genes (0.92% of all expressed genes) in common with both inhibitor-sensitive genes (Additional file 7: Fig. S6J). Notably, 8.0% of the JQ1-sensitive genes were downregulated by the Stattic treatment (110 out of 1373 genes) while 35.3% of the Stattic-sensitive genes were downregulated by JQ1 treatment (110 out of 312 genes; Additional file 7: Fig. S6J, K). Moreover, we found that treatment with JQ1 greatly decreased the fold change in the expression of Stattic-sensitive genes (− 4.0615) compared to that of the Stattic-insensitive genes (− 2.9119; Additional file 7: Fig. S6L). This result suggests that BET proteins can preferentially transactivate Stattic-sensitive genes compared to Stattic-insensitive genes. Collectively, these findings suggest that although BET proteins are essential for the expression of larger subsets of target genes than are downregulated by Stattic, several BET-target genes are downregulated by Stattic.

To further compare the characteristics of the three groups: both inhibitor-sensitive genes (110 genes), JQ1-insensitive but Stattic-sensitive genes (202 genes), or Stattic-insensitive but JQ1-sensitive genes (1263 genes), we conducted Gene Ontology (GO) analysis (Additional file 7: Fig. S6J; Additional file 10: Fig. S7). Functional annotation revealed that both types of inhibitor-sensitive genes were enriched for the GO term associated with “stem cell population maintenance” compared to JQ1-sensitive but Stattic-insensitive genes: 5.5% (6/110) versus 2.1% (26/1263), respectively (Additional file 10: Fig. S7A). However, in terms of “stem cell population maintenance,” we found no association (0%, 0/202) was evident for Stattic-sensitive but JQ1-insensitive genes. We also found genes associated with the GO term linked to “stem cell population maintenance” within both inhibitor-sensitive genes related to six genes, including *Nanog*, *Sox2*, and *Pramel7* (*p*-value = 7.07E−04; Additional file 10: Fig. S7A). However, JQ1-sensitive but Stattic-insensitive genes were associated with the GO term, “stem cell population maintenance,” which related to 26 genes, including *Prdm14*, *Lif*, *Tet1*, *Yap1*, *Tead3*, and *Tead4*, that were previously suggested to be associated with BET proteins (*p*-value = 1.28E−06; Additional file 10: Fig. S7A) [18, 35, 36]. These analyses of target gene identification suggest that other signaling pathways might be involved in the formation of the epiblast lineage via BET proteins. Collectively, they suggest that BET proteins might contribute to later specification and maintenance of the epiblast lineage through Stattic-sensitive and Stattic-insensitive targets (see the “Discussion” section).

### BRD4 is essential for correct maintenance but not for specification of the epiblast lineage

**Fig. 6:**
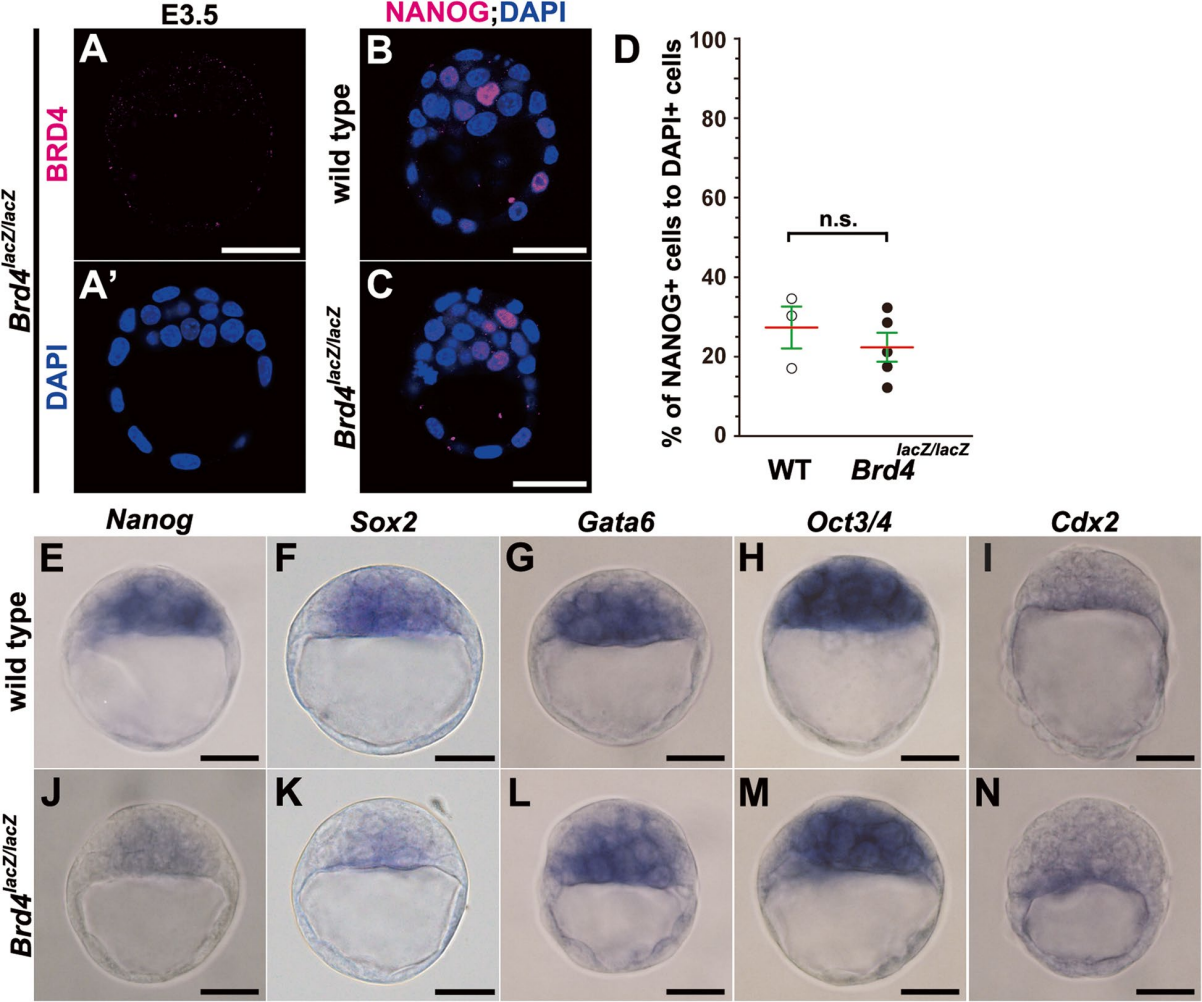
The epiblast lineage can be specified in *Brd4-*deficient blastocysts (stage 3). **A**, **A’** Immunohistochemical analysis of BRD4 protein (magenta; **A**), and DAPI (nuclei, blue; **A’**) staining, in the *Brd4*^*lacZ/lacZ*^ embryos at E3.5. The expression of BRD4 protein is undetectable in a *Brd4*^*lacZ/lacZ*^ embryo. **B**, **C** Immunohistochemical analysis of NANOG (magenta), with DAPI (blue) staining, in wild type (WT) (**B**) or *Brd4*^*lacZ/lacZ*^ E3.5 blastocysts (**C**). NANOG expression was not reduced in a *Brd4*^*lacZ/lacZ*^ blastocyst. **D** The ratio of numbers of NANOG-expressing cells to DAPI-positive cells (nuclei) in WT and *Brd4*^*lacZ/lacZ*^ E3.5 blastocysts (two-tailed Mann**-**Whitney’s *U*-test; n.s., not significant; *p* = 0.571). Red lines indicate the mean values, and green lines represent the SE bars. **E**–**N** Whole-mount in situ hybridization analysis in WT (**E**–**I**) and *Brd4*^*lacZ/lacZ*^ E3.5 blastocysts (**J**–**N**). The expression of *Nanog* (**E**, **J**), *Sox2* (**F**, **K**), *Gata6* (**G**, **L**), *Oct3/4* (**H**, **M**), or *Cdx2* (**I**, **N**) mRNAs. Individual values of marker-expressing cells are provided in Additional file 15. The sample numbers analyzed for each experiment are indicated in Additional file 19. Scale bars, 25 μm in **E**–**N**; 40 μm in **A**–**C**, **A’**

Considering that JQ1 binds to the first bromodomain of BRD4 with the highest affinity, and selectively binds to the second bromodomain of BRD4 as well as to the first and second bromodomains of BRD2 and BRD3 [22], it remains unclear which BET proteins are involved in the formation of the epiblast lineage. To identify the roles of *Brd4* during preimplantation development, we exploited *Brd4* null mutant mice, a *Brd4–lacZ* line in which the genomic region between two *loxP* sites flanking the 5th exon was deleted by crossing with *β-actin Cre* mice [37]. Since BRD4 protein is maternally expressed in oocytes [38], we investigated whether maternally expressed proteins were degraded and lost in *Brd4*-deficient blastocysts. We verified that neither maternal nor zygotic BRD4 products were detectable at E3.5 in *Brd4*^*lacZ/lacZ*^ blastocysts (Fig. 6A, A’), indicating the absence of functional protein in these blastocysts.

Given that JQ1 blocked specification and maintenance of ICM and epiblast lineages in preimplantation development (Figs. 1, 2, 3, and 4; Additional files 1, 2 and 3: Fig. S1–3), we analyzed lineage-specific markers in *Brd4*^*lacZ/lacZ*^ blastocysts at E3.5 (stage 3) (Fig. 6B–N). Notably, neither NANOG protein expression was expressed nor the ratio of the number of NANOG-positive to DAPI-positive cells was decreased in *Brd4*^*lacZ/lacZ*^ blastocysts (Fig. 6B–D). At the mRNA level, however, *Nanog*, as well as *Sox2* expression, tended to be reduced but not diminished in *Brd4*^*lacZ/lacZ*^ blastocysts while the expression of *Gata6*, *Oct3/4*, and *Cdx2* mRNAs was apparently unchanged as expected (Fig. 6E–N). These expression studies indicate that BRD4 is not absolutely necessary for the specification of the epiblast lineage in blastocysts (stage 3) although BRD4 might be necessary for proper expression of epiblast markers at the mRNA level.

To specifically explore the role of *Brd4* in the maintenance of the epiblast lineage (stage 4), we examined the cell lineage-specific markers in *Brd4*-deficient blastocysts at E4.25 (Additional file 11: Fig. S8A–E). In wild-type blastocysts, NANOG-positive epiblast cells were surrounded by a CDX2-positive TE layer and a GATA6-positive PrE layer; PrE cells were polarized and formed epithelium along with a blastocyst cavity (Additional file 11: Fig. S8A, C–C””). In *Brd4*^*lacZ/lacZ*^ blastocysts, however, the number of NANOG-positive cells was decreased inside CDX2-positive TE cells (Additional file 11: Fig. S8B, D–D””). Additionally, GATA6-positive PrE cells appeared to get closer to the outer TE side in place of NANOG-positive epiblast cells but failed to form a single epithelial layer correctly (Additional file 11: Fig. S8B, D–D””). Concurrently, the total cell numbers of ICM-derived inner cells, except the TE layer, had decreased to less than half in *Brd4*^*lacZ/lacZ*^ blastocysts (Additional file 11: Fig. S8F). A cell proliferation marker, phospho-histone H3 (Ser10), was downregulated, and an apoptosis marker, cleaved caspase-3, was upregulated in *Brd4*^*lacZ/lacZ*^ blastocysts (Additional file 11: Fig. S8H–M). Accordingly, the number of NANOG-positive cells in *Brd4*^*lacZ/lacZ*^ blastocysts was greatly reduced, and the ratio of the number of NANOG-positive cells within inner cells was decreased as compared to E4.25 wild-type blastocysts (Additional file 11: Fig. S8E, G). Consequently, the ratio of GATA6-positive cells was increased compared to that of wild-type embryos although the total number of GATA6-positive cells was also decreased to approximately half compared to that of wild-type embryos (Additional file 11: Fig. S8E, G). Such abnormalities of PrE observed in E4.25 *Brd4*^*lacZ/lacZ*^ blastocysts (stage 4) might be due to a failure in the reduced number of epiblast cells after E3.5 blastocysts (stage 3) since a reciprocal interaction between NANOG- and GATA6-positive cells in the ICM is important for correct PrE formation, as previously proposed [1–4, 8]. Together, these findings support the idea that *Brd4* plays an important role in maintaining the epiblast lineage during the blastocyst stage (stage 4).

### BRD2 complements BRD4 function in the specification of the epiblast lineage

Given that JQ1 completely abolished the NANOG-positive epiblast lineage throughout preimplantation development (Figs. 1, 2, 3, and 4; Additional file 2: Fig. S2), the aforementioned *Brd4*-deficient phenotypes observed in E3.5 blastocysts (stage 3) suggest that other BET proteins may play a complementary role in BRD4 in the formation of the epiblast lineage. To explore this redundancy among BET proteins, BRD2 protein expression was analyzed since *Brd2* transcripts displayed a twofold higher level than *Brd3* transcripts (Additional files 4 and 8), and BRDT was reported to be specifically expressed in the testis [39]. Indeed, BRD2 protein was ubiquitously expressed in all nuclei as early as at the 16-cell stage until E4.5 (Additional file 12: Fig. S9A–D’). Then, to evaluate if BRD2 could contribute to the specification of the epiblast lineage, we analyzed *Brd2*^*tg/tg*^ (*Brd2*-deficient) embryos with *Nanog* expression in E3.5 blastocysts (stage 3; Additional file 12: Fig. S9E, E’, F–J) [40]. However, *Nanog* mRNA as well as protein expression appeared to be unchanged in *Brd2*-deficient blastocysts (Additional file 12: Fig. S9F–I). Accordingly, the ratio of the number of NANOG-positive to DAPI-positive cells was not reduced in *Brd2*-deficient blastocysts at E3.5 (stage 3; Additional file 12: Fig. S9J). These expression studies demonstrate that BRD2 alone is dispensable for the specification of the epiblast lineage (stage 3). This finding is also consistent with our previous observation that *Brd2*-deficient embryos are able to survive until E12.5 [40].

**Fig. 7:**
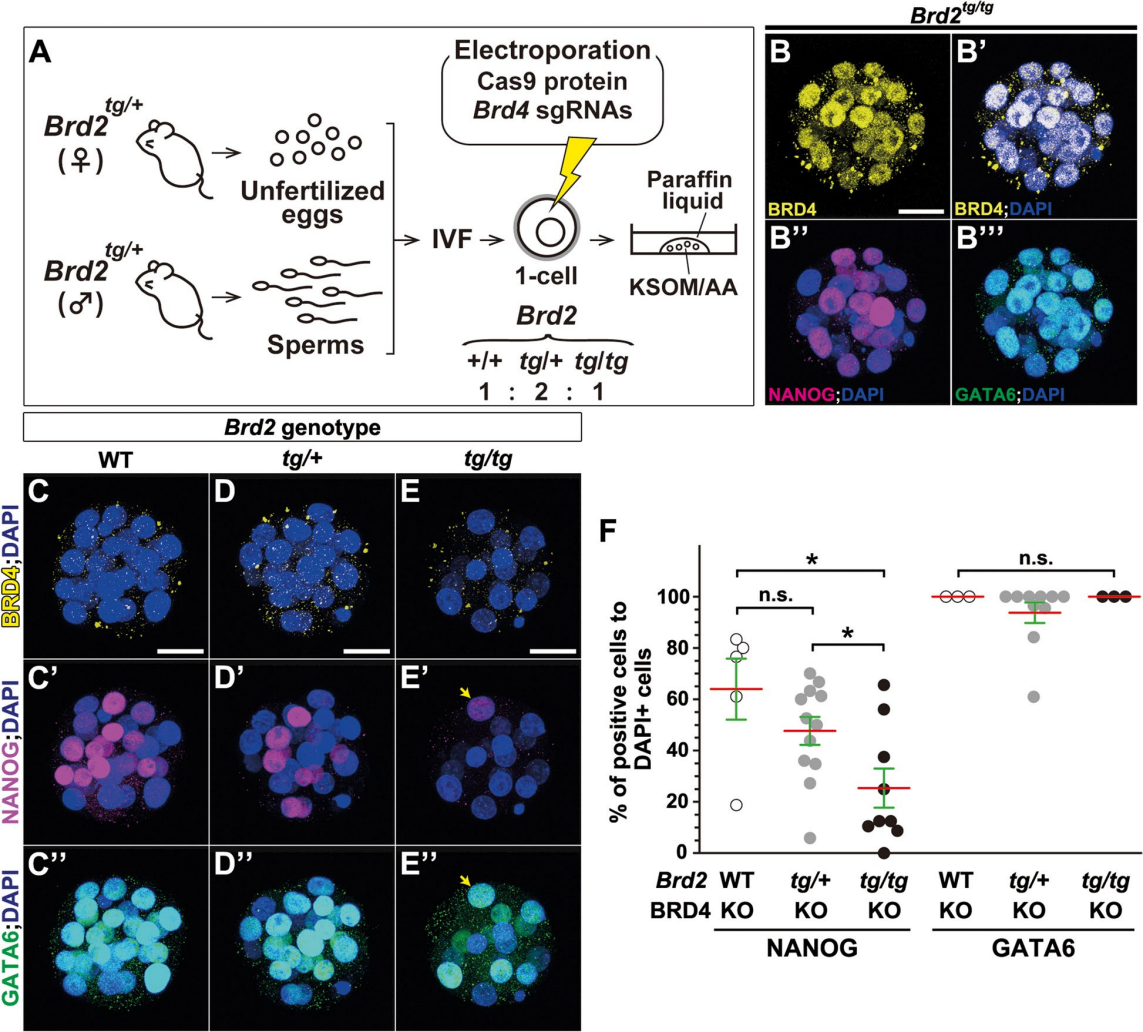
NANOG expression is diminished in *Brd2*^*tg/tg*^; *Brd4* knockout morulae (stage 2). **A** Schematic illustration of the strategy for the generation of *Brd2*^*+/+*^; *Brd4-*, *Brd2*^*tg/+*^; *Brd4-*, and *Brd2*^*tg/tg*^; *Brd4*-knockout embryos with a CRISPR-Cas9 system. **B**–**E**, **B’**, **B”**, **B”’**, **C’**, **C”**, **D’**, **D”**, **E’**, **E”** Immunohistochemical analysis of BRD4 (**B**, **B’**, **C**–**E**; yellow), NANOG (**B”**, **C’**, **D’**, **E’**; magenta), and GATA6 protein (**B”’**, **C”**, **D”**, **E”**; green), with DAPI (nuclei, blue) staining, in *Brd2*^*+/+*^ (**C**, **C’**, **C”**), *Brd2*^*tg/+*^ (**D**, **D’**, **D”**), and *Brd2*^*tg/tg*^ (**B**, **B’**, **B”**, **B”’**, **E**, **E’**, **E”**) late morulae at 16- to 31-cell stages treated with (**C**–**E**, **C’**, **C”**, **D’**, **D”**, **E’**, **E”**) or without (**B**, **B’**, **B”**, **B”’**) electroporation of CAS9 protein and *Brd4* single guide (sg)RNAs. BRD4, NANOG, and GATA6 expression were normally found in *Brd2*^*tg/tg*^ late morulae (**B**, **B’**, **B”**, **B“’**). The remaining NANOG-positive cell still expresses GATA6 (yellow arrows, **E’**, **E”**). **F** The ratio of numbers of NANOG- and GATA6-expressing cells to DAPI-positive cells (nuclei) in late morulae (Kruskal-Wallis test for two markers followed by Steel’s test for NANOG expression; NANOG in *Brd2*^*+/+*^ vs. *Brd2*^*tg/+*^, *p* = 0.092; GATA6 in *Brd2*^*+/+*^ vs. *Brd2*^*tg/+*^vs. *Brd2*^*tg/tg*^, *p* = 0.232; *p < 0.05; n.s., not significant). Red lines indicate the mean values, and green lines represent the SE bars. Individual values of marker-expressing cells are provided in Additional file 15. The sample numbers analyzed for each experiment are indicated in Additional file 19. Scale bars, 25 μm in **B**–**E**, **B’**, **B”**, **B”’**, **C’**, **C”**, **D’**, **D”**, **E’**, **E”**. IVF, in vitro fertilization; WT, wild type; KO, knockout

Next, to explore the redundancy between BRD4 and BRD2 for the specification of the epiblast lineage, we exploited a CRISPR–Cas9 nuclease system to generate efficient transient production of biallelic knockout mice [41]. Since *Brd4* and *Brd2* genes are closely positioned approximately 1.7 Mb apart on the same chromosome (Chr 17), intercrossing of *Brd2*^*tg/+*^ and *Brd4*^*lacZ/+*^ mutant mice to obtain double homozygous mutant embryos would appear to be highly challenging. We then knocked out the *Brd4* gene (*Brd4*-knockout) with CAS9 protein and *Brd4* single guide (sg)RNAs at the 1-cell stage in zygotes from *Brd2*^*tg/+*^ intercrossed mice, and subsequently cultured genome-edited blastomeres (Fig. 7A, Additional file 12: S9K). Since NANOG and GATA6 start to be expressed after the 8-cell blastomere stage and the culture of embryos beyond the 32-cell stage (stage 3) was technically difficult under our experimental conditions (Additional file 12: Fig. S9K) and to evaluate the complementary role of BRD2 for BRD4 in stage 2, we collected late morulae at the 16- to 31-cell stage (stage 2) and examined the expression of NANOG and GATA6 together with BRD4 protein expression (Fig. 7B–F).

In *Brd2*-deficient morulae (stage 2), BRD4 as well as NANOG and GATA6 protein expression were normally detected (Fig. 7B, B’, B”, B”’). After electroporation of CAS9 and *Brd4*-sgRNAs in *Brd2*^*+/+*^*; Brd4*-knockout morulae (stage 2), NANOG and GATA6 protein expression was not reduced reflected by those of conventional *Brd4*^*lacZ/lacZ*^ embryos (Fig. 7C, C’, C”). However, in *Brd2*^*tg/tg*^*; Brd4*-knockout morulae, NANOG expression was diminished while GATA6 expression was not (Fig. 7E, E’, E”). Importantly, removal of one copy of the *Brd2* gene tended to reduce NANOG expression but not GATA6 expression in *Brd4*-knockout morulae (Fig.7D, D’, D"). Accordingly, the ratio of the number of NANOG-positive cells in *Brd2*^*tg/tg*^*; Brd4*-knockout morulae was reduced to half compared to that of *Brd2*^*+/+*^*; Brd4*-knockout morulae (Fig. 7F). In contrast, the ratio of GATA6-positive cells was unchanged in *Brd2*^*tg/tg*^*; Brd4*-knockout morulae compared to that of *Brd2*^*+/+*^*; Brd4*-knockout morulae (Fig. 7F). These expression studies demonstrate that BRD2 is able to complement BRD4 function redundantly in the specification of the ICM lineage for the epiblast formation during preimplantation development. These findings together suggest that the phenotype of JQ1-treated embryos appears to be reflected by *Brd2*^*tg/tg*^*; Brd4*-knockout morulae, rather than by *Brd2*^*tg/+*^*; Brd4*- or *Brd2*^*+/+*^*; Brd4*-knockout morulae.

## Discussion

To date, the BET protein-mediated regulatory mechanisms underlying the formation of the epiblast lineage in mammalian preimplantation have been unknown. Our studies delineate that BET proteins are essential for both specification and maintenance of the epiblast lineage in mouse morulae and blastocysts by activating core transcription factors. In addition, among BET proteins, BRD4 plays a pivotal role and BRD2 plays a complementary role in the specification of the ICM and epiblast lineages. These findings provide unique insights into the roles of BET proteins in the specification and maintenance of the pluripotent epiblast lineage.

### BET proteins play crucial roles in activating gene transcription for the specification and maintenance of the epiblast lineage

BET proteins are necessary for both specification and maintenance of the epiblast lineage in the preimplantation embryo but not for the formation of the TE and PrE directly. BRD4 and BRD2 proteins already start to express from 8-cell blastomeres and JQ1 treatment reduces NANOG expression but does not affect GATA6 expression (stages 1–4; Figs. 2, 3, and 6, Additional file 2: Fig. S2; Additional file 12: Fig. S9). Notably, crucial roles in the formation of the ICM and epiblast lineages by BET proteins are also supported by *Brd2/Brd4* double knockout phenotypes and JQ1-treated embryos whereby total numbers of NANOG-positive cells are diminished in late morulae (Figs. 3 and 7). Considering that ICM cells are specified in stage 2, epiblast cells are specified in stage 3 and maintained in stage 4 (Additional file 1: Fig. S1A) [1–4], and that JQ1 treatment diminishes NANOG-positive epiblast cells in these three stages (Figs. 2 and 3, Additional file 2: Fig. S2), we propose that BET proteins can contribute to both the specification and maintenance of the ICM and epiblast lineages. Consistent with this idea, after JQ1 treatment, NANOG-negative cells were unable to express GATA6; however, NANOG- and GATA6-double-negative cells emerged (Fig. 4). Interestingly, such double-negative cells appear to have retained the competency of the epiblast lineage, even after JQ1 treatment, since these blastocysts were able to re-induce NANOG expression after removal of JQ1 (Fig. 4). Finally, JQ1 treatment and *Brd4-*single or *Brd2;Brd4-*double deficiency have little direct influence on the two extraembryonic lineages, TE and PrE, but, rather, affect these two lineages in a non-cell autonomous manner due to the lower number of epiblast cells (Figs. 2, 3, and 7; Additional file 11: Fig. S8). These several lines of evidence that have been outlined support the notion that BET proteins contribute to both specification and maintenance of the ICM and epiblast lineages primarily in preimplantation development.

Among BET proteins, BRD4 plays a pivotal role in the formation of the epiblast lineage while BRD2 plays a rather complementary role in BRD4 function. NANOG-positive epiblast cells are reduced in *Brd4*-deficient embryos at E4.25 while these cells are not affected in *Brd2*-deficient embryos (Fig. 7, Additional files 11 and 12: Figs. S8, S9). Removal of one copy of the *Brd2* gene in *Brd4*-knockout embryos tended to decrease NANOG-positive cells in late morulae (stage 2; Fig.7). Furthermore, removal of two copies of the *Brd2* gene appears to have decreased the ratio of the number of NANOG-positive cells but not GATA6-positive cells (Fig. 7). Concurrently, *Brd4*-deficient embryos exhibit much earlier defects than *Brd2*-deficient embryos; specifically, *Brd2*-deficient embryos display embryonic lethality around E12.5 and cranial neural tube closure defects [40] while *Brd4*-deficient embryos display severe morphological defects as early as E4.25 (Additional file 11: Fig. S8) and die just after implantation [21]. However, the phenotype of JQ1-treated morulae appears to be severer than those of *Brd2-* and *Brd4*-double knockout morulae (stage 2). The presence of BRD4 and BRD2 maternal proteins might restore BET function in double-KO early morulae. Alternatively, or additionally, we cannot exclude the possibility that other BET proteins, including BRD3, might still have a complementary role in early morulae development. These lines of evidence together support the presence of redundant roles among BET proteins, i.e., the central role of BRD4 and the supportive role of BRD2 in the formation of the ICM and epiblast lineages.

The present study shows that BET proteins are necessary for the transcriptional activation of genes, such as *Nanog*, *Otx2*, *Sox2*, and *Pramel7*, to form the epiblast lineage in mouse preimplantation embryos (Fig. 1, Additional file 3: Fig. S3). Our microarray and RNA-seq analysis showed that many stem cell maintenance genes were downregulated by JQ1 treatment (Additional file 10: Fig. S7; Additional files 4, 8, and 9). These genes are important for maintaining and establishing pluripotent cells in vitro and in vivo [3, 7, 26, 42–45]. In fact, such BET-target genes are mostly expressed in ICM cells (Fig. 1, Additional file 3: Fig. S3; Additional files 4 and 8). Consistently, in *Brd4*-deficient blastocysts at E3.5, *Nanog*, and *Sox2* mRNA expression appeared to be reduced (Fig. 6). In contrast, JQ1 treatment apparently had little impact on the transcriptional activation of genes for the formation of two extraembryonic lineages, TE and PrE (Additional files 4 and 8; Fig. 1; Additional file 3: Fig. S3C; Additional file 10: Fig. S7A). Our comprehensive RNA profiles in JQ1-treated blastocysts further support our hypothesis that BET proteins are crucial to the activation of gene transcription for the formation of the epiblast lineage but not for extraembryonic TE and PrE lineages (Additional files 4 and 8).

In this study, *Oct3/4* mRNA and protein appear to be unaffected by JQ1 treatment as well as *Brd4* deficiency during preimplantation development (Figs. 1, 2, 3, and 6; Additional file 2: Fig. S2C, F; Additional files 4 and 8). In contrast, a significant reduction in *Oct3/4* expression has been reported in mouse ES cells after a 48-h culture with JQ1 treatment [18, 30]. Given that *Oct3/4* expression is known to be activated by transcription factors such as NANOG and SOX2 [45], an immediate reduction of NANOG and SOX2 expression after JQ1 treatment might indirectly diminish transcription of the *Oct3/4* gene in ES cells during a 48-h culture. Consistent with this assumption, prolonged JQ1 treatment of blastocysts for 18 h was able to diminish OCT3/4 expression as well as NANOG and SOX2 (Additional file 2: Fig. S2H–M). These lines of evidence are in good agreement with a recent report that BET proteins activate *Oct3/4* target genes for the pluripotent stem cell lineage, cooperating with OCT3/4 in ES cells [30].

### BET proteins might be crucial to the transactivation of genes partly associated with the STAT3-dependent pathway

Here, we found that BET proteins have transcriptional downstream targets of genes that are directly and indirectly shared with Stattic-sensitive genes (Fig. 5; Additional files 7 and 10: Figs. S6, S7; Additional file 8). We showed that more than one-third of the downregulated genes by Stattic appeared to be BET targets, although the remaining targets were not affected by BET inhibition (Additional file 7: Fig. S6J, K). Moreover, JQ1-sensitive genes among Stattic-sensitive genes were more downregulated than among Stattic-insensitive genes (Additional file 7: Fig. S6L). Concurrently, both JQ1- and Stattic-sensitive genes, such as *Nanog*, *Sox2*, and *Pramel7*, are apparently crucial to the maintenance of the epiblast lineage in terms of their expression and functions in ES cells [7, 42–45]. STAT3 has been shown to be crucial in maintaining the pluripotent state of ES cells as well as ICM formation in blastocysts [10, 46–48]. *Stat3*-deficient blastocysts appear to form three lineages, i.e., TE, epiblasts, and PrE, normally until E3.5 but fail to maintain epiblast and PrE lineages after E4.0 (stage 4) [10]. Although further precise analyses will be required to clarify the similarities and differences between *Brd4-* and *Stat3*-deficient blastocysts, STAT3 may partly contribute to the maintenance of epiblast and PrE lineages cooperatively with BRD4 but not to specification. Therefore, BET proteins might be involved in STAT3-mediated transcriptional activation for the maintenance of the epiblast lineage but not primarily its specification.

Mechanistically, canonical JAK/STAT signaling underlies a molecular mechanism that begins with extracellular ligands and the transcriptional activation of their downstream targets [48, 49]. This pathway is activated by the interleukin-6 (IL-6) family of cytokines, such as IL-6 and LIF, through multimerization of receptors, such as gp130 and the LIF receptor. Thereafter, the receptors’ activation is intracellularly transmitted to JAK tyrosine kinases, and finally, phosphorylated STAT binds *cis*-regulatory regions and activates transcription of target genes in the nucleus [48, 49]. Phosphorylation of nuclear STAT3 is diminished by JQ1 treatment and in *Brd4*^*lacZ/lacZ*^ blastocysts (Additional file 6: Fig. S5B–D’). Considering that excess LIF was not able to complement nor overcome adverse effects due to JQ1 treatment in blastocysts (Additional file 6: Fig. S5H–M), this means that BET proteins might also be located downstream of LIF and cooperatively act with STAT3 in the nucleus for transcriptional activation. Therefore, one possibility is that BET proteins might be involved in the phosphorylated state of STAT3, and thus, phosphorylation of STAT3 may occur by recruiting P-TEFb kinase indirectly or by direct kinase activity of BRD4 itself as previously reported [12, 13, 50]. Consistent with such a hypothesis, BRD4 can form a protein complex with STAT3 and control transcription in other cell lines [51, 52].

Alternatively, non-canonical JAK/STAT signaling might be involved in the transcriptional activation of the *Nanog* gene [33, 34]. JAK2 can modulate (causative) heterochromatin directly in a LIF-independent manner and be specifically blocked by TG101209 [34]. As unphosphorylated STAT is localized on heterochromatin in association with heterochromatin protein 1 in the nucleus, phosphorylation of STAT reduces the total amount of heterochromatin-associated STAT and consequently leads to heterochromatin instability [33]. Therefore, it can be hypothesized that the switch between euchromatin and (causative) heterochromatin can also contribute to JQ1- and/or Stattic-mediated transcriptional inactivation in the present study. To address which molecular mechanisms are involved in the maintenance of the epiblast lineage, further intensive studies on the transcriptional machinery of BRD4, RNA polymerase II, STAT3, histone acetylation, and methylation will be necessary.

In this study, we utilized Stattic at a 1.0 μM concentration, which is lower than that of the IC50 for STAT3 at a 5.1 μM concentration as demonstrated in the previous cell-free assay [32]. To analyze the cell fate of each single blastomere in mouse preimplantation embryos with lineage-specific markers at the protein level, we have considered the half-life of proteins, i.e., the time lag in degradation of endogenous protein products because it has been shown that the half-life of *Nanog* mRNA is about 4 h [53] and that of NANOG protein is more than 4 h [54]. Thus, to avoid severe developmental retardation by 6-h Stattic treatment (Additional file 5: Fig. S4F, F’), searching for a viable condition after 6 h of culture was necessary (Additional file 5: Fig. S4A-G). Although we have verified that the effects on the expression of pSTAT3 and some of the target genes with 1.0 μM Stattic appear to show a similar tendency to those using 5.0 μM Stattic (Fig. 5, Additional file 5: Fig. S4), we cannot exclude the possibility that Stattic might affect gene transcription through an off-target effect but not through STAT3 inhibition directly. To verify this, a further comparative expression study with RNA-seq data from maternal and zygotic *Stat3*-deficient embryos will be essential.

With the exception of the Stattic-sensitive pathway, BET proteins appear to activate transcriptional targets to maintain the epiblast lineage. Our expression studies indicated that of these JQ1-sensitive genes, only 8% are common to Stattic-sensitive genes and 92% involve Stattic-insensitive targets (Additional file 7: Fig. S6K). These genes include *Prdm14*, *Sall1*, *Hes1*, *Fgfr3*, *Piwil2*, *Nodal*, *Tet1*, *Tead3*, and *Yap1*, among others (Additional file 10: Fig. S7A). A reduction of such genes might also suggest the involvement of Hippo and FGF signaling, in the specification and/or maintenance of an epiblast lineage by BET proteins. To address whether BET proteins regulate Hippo and FGF signaling pathways for the formation of the epiblast lineage, further molecular and genetic studies will be required.

## Conclusion

From this study, we conclude that BET proteins contribute to transcriptional activation for both the specification and maintenance of the epiblast lineage. Among BET proteins, BRD4 plays a pivotal role and BRD2 plays a complementary role in BRD4 function during specification of the epiblast lineage in the development of mouse preimplantation. Additionally, BET-dependent maintenance of the epiblast lineage may be partly associated with the STAT3-dependent pathway.

## Methods

### Mutant mice

To obtain *Brd4*^*lacZ/+*^ mice, in vitro fertilization was performed using the sperm of a heterozygous *Brd4*^*tm1a(EUCOMM)Wtsi*^ mouse (obtained from the Mutant Mouse Resource and Research Center [MMRRC]), and the oocytes of a *β-actin Cre* mouse [37]. In vitro fertilized oocytes were transferred to pseudo-pregnant recipients according to the standard methods [55]. The resulting *Brd4*^*lacZ/+*^ mice were genotyped using the following three primers: Brd4-wt_for (5′-CCTGTTTCCTCATCCAGCCTGGAGATGACA-3′), Brd4-lacZ_for (5′-GCAACTGATGGAAACCAGCCATCGCCATCT-3′), and Brd4_rev (5′-GCAGGACCAGGGCATGAAGCTCAATGGTGA-3′), yielding 521 bp as the wild-type allele and 646 bp as the *Brd4*^*lacZ*^ allele. For genotyping of *Brd4*^*lacZ/lacZ*^ embryos at E3.5 and E4.25, first and second rounds of PCR were performed with the following primers: Brd4-1st_for (5′-CTCAGCTTTTGACCTCTGCTCGTGTAGTGG-3′), Brd4-wt-1st_rev (5′-CCTAACGGGAGGCCCTCCTTTACTTTGGGA-3′), and Brd4-lacZ-1st_rev (5′-CTGTTAGTCCCAACCCCTTCCTCCTACATA-3′) for first PCR, and Brd4-2nd_for (5′-CAGACTAGGCCCTCAGTTTAGAGCTGAACG-3′), Brd4-wt-2nd_rev (5′-CCAACAGTGCTCCCTAATGTACCCCAATGG-3′), and Brd4-lacZ-2nd_rev (5′-GTTGGCAGTGTTTGGGGCAAGTGTGGAGGG-3′) for second PCR, yielding 144 bp as the wild-type allele and 248 bp as the *Brd4*^*lacZ*^ allele. All mice were maintained in a CD-1 background.

*Brd2*^*tg/+*^ mice were generated as described previously [40] and maintained in a CD-1 background. For genotyping of *Brd2*^*tg/tg*^ embryos at morula and blastocyst stages, first and second rounds of PCR were performed with the following primers: Brd2-wt-1st_for (5′-TGCGCAGATCCAGCCTTGATTTGTCATCGT-3′), Brd2-tg-1st_for (5′-TATCATGTCTGGATCCCCAGGAAGCTCCTC-3′), and Brd2-1st_rev (5′-CCAGGTGTTTCCTAATGGCTAAGACACTAG-3′) for the first round of PCR, and Brd2-wt-2nd_for (5′-TAAGACCTTCAACGGCAGCCTGTACCTATG-3′), Brd2-tg-2nd_for (5′-GGAAGTCCCTTCCACTGCTGTGTTCCAGAA-3′), and Brd2-2nd_rev (5′-AGTTGCCATGGTGACAGCTTGAGGGTTTGC) for the second round of PCR, yielding 116 bp as the wild-type allele and 166 bp as the *Brd2*^*tg*^ allele. The *Brd2*^*tg/+*^ mice were genotyped with the same primers as used in the first round of PCR, yielding 329 bp as the wild-type allele and 428 bp as the *Brd2*^*tg*^ allele.

### Embryonic culture and treatment with chemical inhibitors and LIF

Embryos from E2.5 to E3.5 (stages 1 to 3) were flushed from the oviduct or uterus of each mouse using KSOM/AA medium. After collected embryos were pre-cultured with KSOM/AA medium for 30 min at 37 °C and 5% CO_2_, they were then cultured with chemical inhibitors or vehicle control at 37 °C and 5% CO_2_. The culture medium contained final concentrations of 5 μM (+)-JQ1 (JQ1) (Cayman, Ann Arbor, MI, USA; dissolved in dimethyl sulfoxide [DMSO]), 1 μM or 5 μM Stattic (Cayman; dissolved in DMSO), 500 nM TG101209 (Cayman; dissolved in DMSO), 5000 units/mL (5×) LIF (Millipore, Burlington, MA, USA; and ESGRO mLIF Supplement dissolved in 1% bovine serum albumin [BSA] in phosphate saline buffer [PBS]). Culture times are described in legends. For 30-h prolonged culture, embryos at E3.5 were flushed out as described above and cultured with 5 μM JQ1 or DMSO for 6 h; thereafter, the culture medium was changed, and embryos were cultured in the presence of 5 μM JQ1 or DMSO for an additional 24 h. For *Brd4*^*lacZ/lacZ*^ embryo culture, embryos at E3.5 were cultured with KSOM/AA medium for 6 h.

### Whole-mount immunohistochemistry

For whole-mount immunohistochemistry, embryos from E2.5 to E4.5 were fixed in 4% paraformaldehyde (PFA) in PBS for 1 h at 4 °C, washed several times in PBS containing 0.1% Triton X-100 (PBST), and permeabilized in PBS containing 0.5% Triton X-100 for 10 min at room temperature (RT). For staining with an antibody against phosphorylated STAT3 protein, embryos were permeabilized in methanol at − 20°C for 10 min. The embryos were incubated with PBST containing 1% BSA and 10% goat or donkey serum for 1 h at RT, then immersed overnight at 4 °C in primary antibody solution diluted in blocking solution as shown in Additional file 13. The embryos were washed eight times in PBST for 10 min per wash and incubated with appropriate species-specific fluorophore-conjugated secondary antibodies (Invitrogen, Carlsbad, CA, USA) for 2 h at RT. For the immunohistochemical staining for the triple staining of NANOG, GATA6, and CDX2, at E4.25, the embryos were fixed in 4% PFA in PBS for 20 min, washed in 3 mg/mL polyvinyl pyrrolidone (PVP) in PBS, permeabilized in PBS containing 3 mg/mL PVP and 0.25% Triton X-100 for 30 min, and blocked in PBS containing 0.1% BSA, 0.01% Tween 20, and 2% goat serum for 15 min, all at RT. The embryos were then immersed overnight at 4 °C in a primary antibody solution diluted in the blocking solution. The embryos were washed three times for 15 min in blocking solution and then incubated with the appropriate secondary antibodies in blocking solution for 2 h at RT. The nuclei were stained with DAPI (Lonza, Morrisville, NC, USA). The embryos were mounted with Vectashield Mounting Medium (Vector Laboratories, Burlingame, CA, USA). Images were examined and captured with Olympus FV1000, FV3000 or Leica TCS SP8 confocal microscopes.

### Cell counts

For cell count analysis, three-dimensional (3D) confocal images were constructed by capturing *Z*-planes every 2.0 or 2.5 μm. For total cell counts, the number of DAPI-positive cells was counted. To count marker-positive cells, “ratio analysis” in the OLYMPUS cellSens Dimension software (version 2.3) was conducted according to the manufacturer’s instructions. Ratio analysis divides one image by another on a pixel-by-pixel basis. In brief, first, the image of the antibody (marker-positive) channel was set as the numerator and that of the DAPI channel was set as the denominator. Second, after removal of the background value, a threshold of intensity fluorescence value for images of each channel for the ratio analysis was set as shown in Additional file 14. Third, a scaling factor was set to 10,000, and ratio analysis was performed; the fluorescence intensity value of marker-positive cells was normalized in the DAPI fluorescence intensity per cell. After ratio analysis, marker-positive cells were distinguished from marker-negative cells using a cutoff value of the average intensity value in the maximum area of the nucleus as shown in Additional file 14 and then counted. Double-positive cells for pSTAT3 and NANOG were decided after each marker-positive cell was counted using ratio analysis. In terms of cleaved caspase-3- and phospho-histone H3-positive cells, since positive cells were clearly distinguishable, ratio analysis was not used; specifically, regarding the cell counting of phospho-histone H3 positive-cells, those cells that displayed a dotted pattern in the late G2 phase and a dividing pattern in M-phase, respectively, were counted.

For the analyses of E4.25 embryos, immunohistochemistry was performed using NANOG, GATA6, and CDX2 antibodies simultaneously. All CDX2-negative cells were counted as ICM cells. The number of NANOG-positive but CDX2-negative cells, GATA6-positive but CDX2-negative cells, NANOG-positive and GATA6-positive cells (double-positive [DP]), and NANOG-negative and GATA6-negative cells (double-negative [DN]) were counted after 3D reconstruction.

Absolute values of marker- and DAPI-positive cell numbers are described in Additional file 15.

### Analysis of intensity values of phosphorylated STAT3

For the analysis of intensity values of pSTAT3, 3D confocal images were constructed by capturing *Z*-planes every 2.5 μm. Then, ratio analysis was conducted as above described in the method of cell counts; a threshold of intensity fluorescence value for images of each channel for the ratio analysis was set as 1000 for pSTAT3 and 500 for DAPI, and the fluorescence intensity value of pSTAT3 was normalized in the DAPI fluorescence intensity per cell (Additional file 16). After the ratio analysis, the top 10 of the average intensity value in the maximum area of the nucleus per embryo were plotted, and their average intensity of blastocysts treated with control (DMSO) and 1 μM or 5 μM Stattic for 3h and 6h was compared.

### Whole-mount in situ hybridization

*Nanog*, *Gata6*, *Oct3/4*, *Sox2*, *Sox17*, and *Cdx2* plasmids for RNA antisense probes for in situ hybridization were gifts from Dr. S. Yamanaka, Dr. F. Grosveld, Dr. H. Schöler, Dr. R. Lovell-Badge, Dr. M. Kanai-Azuma, and Dr. J. Robert, respectively. The *Otx2* plasmid for the in situ RNA probe was generated as described [56]. Other plasmids for RNA probes (*Arid5b*, *Pramel6*, *Pramel7*, *Trim43a*) were constructed as follows: PCR products were amplified from mouse cDNA or a C57BL/6 genome and subcloned into pTAC-2. Primer sets for PCR amplification and their respective lengths are shown in Additional file 17. In situ hybridization involving digoxigenin-labeled probes was conducted in a manner identical to that of Wilkinson [57].

### RNA-FISH

Mouse blastocysts at E3.5 were cultured in KSOM/AA medium containing DMSO or 5 μM JQ1 for 1 h at 37 °C and 5% CO_2_. The cultured embryos were then treated with acidic Tyrode’s solution (Sigma, St Louis, MI, USA) to remove their zona pellucida, washed several times in M2 medium, and incubated in PBS containing 6 mg/mL BSA for 5 min at RT. The embryos were transferred onto a glass slide coated with Denhardt’s solution and dried down for 15 min at RT. The slides were fixed and permeabilized simultaneously in ice-cold 1% PFA in PBS containing 0.5% of TERGITOL™ solution Type NP-40, 70% in H_2_O (Sigma) for 5 min to reduce the cytoplasmic background, sequentially fixed in ice-cold 1% PFA in PBS for 5 min, and then stored in 70% ethanol (EtOH) at − 20 °C until use. Prior to RNA fluorescence in situ hybridization (FISH), the slides were dehydrated in a standard EtOH series (70%, 80%, and 100% EtOH, each two times for 2 min at RT) and dried for 5 min at RT. The slides were then hybridized overnight at 37 °C with 200 ng of RNA-FISH probe and 10 μg of herring sperm DNA (Invitrogen), 20 μg of mouse Cot-1 DNA (Invitrogen), 20 μg of yeast tRNA (Roche, Basel Switzerland), and 20 mM ribonucleoside vanadyl complex (New England Biolabs, Ipswich, MA, USA) in 20 μL of hybridization buffer (50% formamide, 2× saline-sodium citrate [SSC; pH 7.0], 2 mg/mL BSA, 10% dextran sulfate) per slide. The slides were washed four times with 50% formamide in 2×SSC followed by washing three times with 2×SSC each for 5 min at 45 °C. The slides were then stained with 25 μg/mL DAPI (Lonza) in 2×SSC for 3 min, washed several times with 2×SSC, and mounted with Vectashield Mounting Medium (Vector Laboratories).

The following bacterial artificial chromosome (BAC) clones were subcloned using a BAC Subcloning Kit, Red/ET Recombination (GeneBridges, Heidelberg, Germany) according to the manufacturer’s instructions and used for the synthesis of the following RNA-FISH probes: *Nanog*, B6Ng01-118J17 (RIKEN BRC, Tsukuba, Japan); *Gata6*, B6Ng01-347A11 (RIKEN BRC); *Oct3/4*, RP23-38P5 (Life Technologies, Carlsbad, CA, USA); and *Cdx2*, B6Ng01-081M23 (RIKEN BRC). Genomic regions for RNA-FISH probes and their lengths are shown in Additional file 18. To generate RNA-FISH probes, templates were labeled with Cy3-dUTP (GE Healthcare, Chicago, IL, USA) using a Nick Translation Kit (Roche). Labeled probes were synthesized for 16 h at 15 °C and purified using an Illustra AutoSeq G-50 Dye Terminator Removal Kit (GE Healthcare) according to the manufacturer’s instructions. All RNA-FISH images were captured with a Leica TCS SP8 confocal microscope.

### Microarray and ingenuity pathway analysis

For microarray analysis, mouse blastocysts at E3.5 (stage 3) were cultured in triplicate with KSOM/AA medium containing DMSO or 5 μM JQ1 for 3 h at 37 °C and 5% CO_2_ (DMSO treatment: *n* = 72, *n* =145, *n* = 112; JQ1 treatment: *n* = 182, *n* = 162, *n* = 204), immersed in TRIzol Reagent (Invitrogen) and stored at − 80 °C until use. Total RNA was extracted from embryos using Micro Smash (TOMY, Japan) and RNeasy Plus Micro Kits (Qiagen, Hilden, Germany) according to the manufacturer’s instructions. The purity of total RNA was assessed using an Agilent 2100 Bioanalyzer and Agilent RNA 6000 Pico Kit (Agilent Technologies, Santa Clara, CA, USA). All total RNA used had an RNA integrity number greater than 8.5. Biotinylated cRNA was prepared from 50 ng total RNA using a GeneChip WT PLUS Reagent Kit (Affymetrix, Santa Clara, CA, USA) and hybridized on a GeneChip Mouse Gene 2.0 ST Array (Affymetrix) according to the manufacturer’s instructions. Chips were washed and stained using a GeneChip Hybridization, Wash, and Stain Kit Fluidics Station 450 (Affymetrix). Arrays were scanned on a GeneChip Scanner 3000 7G (Affymetrix) and analyzed first with a GeneChip Command Console (Affymetrix). The raw data of each set of three arrays were then analyzed separately and normalized using an Affymetrix Expression Console (Affymetrix). Differentially expressed genes were identified using an Affymetrix Transcriptome Analysis Console (Affymetrix) for those showing a ≥ 2.0-fold change in expression between samples from embryos treated with DMSO and JQ1, with an adjusted *p*-value < 0.05. Hierarchical clustering analysis was performed using GeneSpring GX 14.9 (Agilent). To investigate the canonical pathways, differentially expressed genes obtained by microarray analysis were subjected to Ingenuity Pathway Analysis (IPA) ver. 36601845 (Ingenuity) [58].

### RNA sequencing

For RNA sequencing, blastocysts at E3.5 were cultured in triplicate with KSOM/AA medium containing DMSO, 5 μM JQ1 or 1 μM Stattic for 6 h at 37 °C and 5% CO_2_ (DMSO treatment: *n* = 61, *n* = 43, *n* = 54; JQ1 treatment: *n* = 66, *n* = 47, *n* = 67; Stattic treatment: *n* = 61, *n* = 86, *n* = 71), immersed in TRIzol Reagent (Invitrogen) and stored at − 80°C until use. Each cDNA was generated using a Clontech SMART-Seq HT Kit (Takara Clontech, Mountain View, CA, USA). Each library was prepared using a Nextera XT DNA Library Prep Kit (Illumina, San Diego, CA, USA) according to the manufacturer’s instructions. Whole transcriptome sequencing was applied to RNA samples on an Illumina HiSeq 2500 platform in a 75-base single-end mode. Sequenced reads were mapped to mouse reference genome sequences (mm10) using TopHat ver. 2.0.13 in combination with Bowtie2 ver. 2.2.3 and SAMtools ver. 1.0. The number of fragments per kilobase of exon per million mapped fragments (FPKMs) was calculated using Cufflinks ver. 2.2.1. Only genes with a FPKM greater than 0.1 in three samples were used for the analysis of differentially expressed genes. The differentially expressed genes were determined by showing a ≥ 2.0-fold change in the expression between treatments, with an adjusted *p*-value < 0.05. The 10th percentile, first quartile, median, third quartile, and 90th percentile were plotted as box-and-whiskers graphs.

### Gene Ontology analysis

Gene Ontology analyses were performed using DAVID ver. 6.8 on genes of the following three groups: genes downregulated by both JQ1 and Stattic treatments (JQ1- and Stattic-sensitive genes), genes downregulated by JQ1 only treatment but not by Stattic treatment (JQ1-sensitive but Stattic-insensitive genes), and genes downregulated by Stattic only treatment but not by JQ1 treatment (Stattic-sensitive but JQ1-insensitive genes; RNA sequence, FPKM ≥ 0.1, fold change ≥ 2.0, *p* < 0.05).

### Generation of Brd2;Brd4-knockout embryos

To obtain *Brd2*^*tg/tg*^ oocytes, unfertilized *Brd2*^*tg/+*^ oocytes were in vitro fertilized with sperm obtained from *Brd2*^*tg/+*^ males. In vitro fertilization (IVF) was performed according to the standard protocol [55]. The covering cumulus cells were removed by 1% hyaluronidase/M2 medium. The fertilized eggs were incubated in KSOM/AA medium until electroporation. For electroporation, a CUY21 EDITII, GE-101 platinum plate electrode (length 10 mm, width 3 mm, height 0.5 mm, gap 1 mm), and GE-1 electrode holder (BEX Co. Ltd., Tokyo, Japan) were used. After IVF, 30–50 zygotes were subjected to electroporation at one time. The collected zygotes cultured in KSOM/AA medium were washed with Opti-MEM I (Thermo Fischer Scientific) three times and placed in a line in the electrode gap filled with 5 μL of Opti-MEM I medium containing 40 ng/μL each of three *Brd4* sgRNAs and 100 ng/μL Alt-R S.p. HiFi Cas9 Nuclease V3 (Integrated DNA Technologies [IDT], Coralville, IO, USA), and subjected to electroporation. The electric conditions were 25 V (3 msec ON + 97 msec OFF) three times in each experiment. After electroporation, the zygotes were immediately collected from the electrode chamber and washed with M2 medium three times followed by two washes with KSOM/AA medium (KYUDO Co., Ltd., Japan). Then, the electroporated zygotes were cultured with KSOM/AA medium covered with paraffin liquid (Nacalai Tesque, Kyoto, Japan) for 75 h at 37 °C and 5% CO_2_. Three *Brd4* sgRNAs were purchased from IDT and guide RNA sequences used were BRD4.1AA (5′-TTTGGTACCGTGGATACACC AGG), BRD4.1AC (5′-GGGGGCACCATTGTCATGAC AGG), and BRD4.1AE (5′-ATGGGCGGGTGACTCTGCAC AGG).

### Animal experiments

All mouse studies followed fundamental ethical guidelines for the proper conduct of animal experiments and related activities in academic research institutions under the jurisdiction of the Ministry of Education, Culture, Sports, Science, and Technology of Japan, and were approved by institutional committees at the Research Institute for Osaka Women’s and Children’s Hospital for animal and recombinant DNA experiments.

### Statistical analysis

Statistical analyses were performed with Mann-Whitney’s *U*-test, Kruskal-Wallis test followed by Steel’s test, and one-way ANOVA followed by the Tukey-Kramer test as described in the legends. The data are presented as the mean ± standard error (SE). Data analysis for Mann-Whitney’s *U*-test was performed using EZR ver. 1.40 (Saitama Medical Center, Jichi Medical University, Saitama, Japan), which is a graphical user interface for R (The R Foundation for Statistical Computing, Vienna, Austria). More precisely, it is a modified version of R commander designed to add statistical functions frequently used in biostatistics [59]. For Kruskal-Wallis test, one-way ANOVA, and post hoc analysis, Bell Curve for Excel ver. 3.21 (Social Survey Research Information Co., Ltd. Tokyo, Japan) was used. The sample numbers analyzed for each experiment are shown in Additional file 19. In all cases, significance was noted at *p < 0.05, **p < 0.01, and ***p < 0.001.

## Supplementary Information

**Additional file 1: Fig. S1.** Schematic illustrations of our experimental strategy and embryonic stages before and after JQ1 treatment in mouse preimplantation development. (A) Schematic illustration of the formation of three cell lineages and expression of lineage-specific markers during mouse preimplantation development. The number of total cells in embryos are depicted on the left side along with the preimplantation stages as described in Results (“Stage 1” to “Stage 4”). (B) Schematic illustration of the experimental strategy for mouse E3.5 blastocyst (45 to 64 cells) culture without or with JQ1. (C–C”) Immunohistochemical analysis of a 56-cell stage E3.5 blastocyst before treatment (Stage 3). NANOG (magenta), GATA6 (green), and DAPI (nuclei, blue) staining; and a merged view without DAPI staining. E3.5 blastocysts (45 to 64 cells) were composed of mostly co-expressing inner cell mass (ICM; white, arrows) and mutually exclusive, salt and pepper ICM (magenta or green) cells in terms of NANOG and GATA6 (C”). (D) The number of total cells (DAPI-positive cells) before, after without JQ1 or with 5 μM JQ1 6 h-treatment of E3.5 blastocysts (two-tailed Mann–Whitney’s *U*-test, n.s.: not significant; *p* = 0.804). Red lines indicate mean values and green lines represent SE bars. Individual values of markers-expressing cells are provided in Additional file 15. The sample numbers analyzed for each experiment are indicated in Additional file 19. Scale bars: 20 μm in C–C”. DMSO, dimethyl sulfoxide; EPI, epiblast; PrE, primitive endoderm; RNA–FISH, RNA–fluorescence *in situ* hybridization; TE, trophectoderm. https://doi.org/10.6084/m9.figshare.19126595

**Additional file 2: Fig. S2.** Expression of lineage-specific markers after JQ1 treatment of morulae (Stage 1) and blastocysts (Stage 4). (A–F) Immunohistochemical analysis of 16-cell stage morulae (Stage 1) treated without JQ1 (control; A–C) or with 5 μM JQ1 (D–F) for 6 h of 8-cell blastomeres. NANOG (A,D), GATA6 (B,E), and OCT3/4 (C,F) (magenta), with DAPI (nuclei, blue) staining. (G) The ratio of numbers of NANOG-, GATA6-, or OCT3/4-expressing cells to DAPI-positive cells (nuclei) treated without JQ1 or with 5 μM JQ1 for 6 h of E2.5 embryos (Stage 1) (two-tailed Mann–Whitney’s *U*-test, ***p < 0.001, n.s.: not significant; GATA6; *p* = 0.483, OCT3/4; *p* = 0.399). Red lines indicate the mean value and green lines represent SE bars. (H–M) Immunohistochemical analysis of NANOG (H,K), SOX2 (I,L), and OCT3/4 (J,M) (magenta), with DAPI (H–M, blue) staining, in E3.5 blastocysts cultured without JQ1 (control) or with 5 μM JQ1 for 18 h (Stage 3 to 4). Individual values of markers-expressing cells are provided in Additional file 15. The sample numbers analyzed for each experiment are indicated in Additional file 19. Scale bars: 40 μm in A–F, H–M. https://doi.org/10.6084/m9.figshare.19134794

**Additional file 3: Fig. S3.** Identification of down-regulated genes in JQ1-treated mouse blastocysts with microarray (Stage 3). (A) Schematic illustration of the experimental method for microarray. (B) Heatmap of the hierarchical clustering of down- and up-regulated genes following treatment without JQ1 (control) or with 5 μM JQ1 for 3 h (>2.0-fold, *p* < 0.05). A red color indicates up-regulated expression, yellow means unchanged expression, and blue indicates down-regulated expression. *n*=3 independent samples for each experimental condition. Color indicates bi-weight average signal (log_2_). (C) List of the top 15 down-regulated genes after JQ1 treatment in mouse blastocysts after microarray analysis. The listed genes are selected among genes having a bi-weight average signal (log_2_) of control blastocysts larger than 8.5. (D–K) Whole-mount *in situ* hybridization of down-regulated (*Arid5b*, *Pramel6, Pramel7*, *Trim43a*) genes identified by microarray analysis of mouse E3.5 blastocysts treated without JQ1 (D–G) or with 5 μM JQ1 (H–K) for 3 h. The sample numbers analyzed for each experiment are indicated in Additional file 19. Scale bars: 25 μm in D–K. DMSO, dimethyl sulfoxide. https://doi.org/10.6084/m9.figshare.19134857

**Additional file 4.** Microarray data in mouse blastocysts. https://doi.org/10.6084/m9.figshare.19134866

**Additional file 5: Fig. S4.** Impact of consideration of Stattic concentration on pSTAT3 expression and JAK2 inhibitor on pSTAT3 expression and NANOG-positive cells (Stages 3 and 4). (A–F’) Immunohistochemical analysis of phosphorylated (p)STAT3 (A–F’, magenta), with DAPI (A’–F’, blue) staining, in mouse E3.5 blastocysts without Stattic (control) (A,A’), or treated with 1 μM (C,C’) and 5 μM Stattic (E,E’) for 3 h and without Stattic (control) (B,B’) or treated with 1 μM (D,D’) and 5 μM Stattic (F,F’) for 6 h. (G) Quantification of intensity values of pSTAT3 expression in ICM of E3.5 blastocysts treated without Stattic (control), or treated with 1 μM and 5 μM Stattic for 3h and 6h (one-way ANOVA followed by Tukey-Kramer test; ***p < 0.001, n.s.: not significant; 1 μM Stattic for 6h *vs.* 5 μM Stattic for 3h, *p* = 1.000; 1 μM Stattic for 6h *vs.* 5 μM Stattic for 6h, *p* = 0.425; 5 μM Stattic for 3h *vs.* 5 μM Stattic for 6h, *p* = 0.692). Red lines indicate mean values and green lines represent SE bars. Individual intensity values of pSTAT3 were provided in Additional file 16. (H–I’) Immunohistochemical analysis of pSTAT3 (H–I’, magenta), with DAPI (H’,I’, blue) staining, in mouse E3.5 blastocysts without TG101209 (control; H,H’), or treated with 500 nM TG101209 (I,I’) for 6 h. (J–M”’) Immunohistochemical analysis of NANOG (magenta) and GATA6 (green), with DAPI (nuclei, blue) staining, in mouse blastocysts (Stage 3 to 4) without TG101209 (control; J–K”’), or treated with 500 nM TG101209 (L–M”’) for 6 h. (N) The ratio of numbers of NANOG- or GATA6-expressing cells to DAPI-positive cells (nuclei) treated without TG101209 or 500 nM TG101209 (TG) for 6 h in E3.5 embryos (two-tailed Mann–Whitney’s *U*-test, **p < 0.01, n.s.: not significant; *p* = 0.537). Red lines indicate the mean value and green lines represent SE bars. Individual values of markers-expressing cells are provided in Additional file 15. The sample numbers analyzed for each experiment are indicated in Additional file 19. Scale bars: 20 μm in K–K”’, M–M”’, 40 μm in A–F’,H–I’J,L. https://doi.org/10.6084/m9.figshare.19134890

**Additional file 6: Fig. S5.** Impact of JQ1 treatment, *Brd4*^*lacZ/lacZ*^ embryos and additional LIF on JQ1 treatment on pSTAT3 expression (Stage 3 to 4). (A–D’) Immunohistochemical analysis of phosphorylated STAT3 (A–D’, magenta), with DAPI (A’ –D’, blue) staining, in mouse E3.5 blastocysts without JQ1 (control) (A,A’), or treated with 5 μM JQ1 (B,B’) for 6 h; and cultured E3.5 blastocysts of wild type (C,C’) and *Brd4*^*lacZ/lacZ*^ embryos (D,D’) for 6 h (Stages 3 and 4). The expression of pSTAT3 shown in here was significantly downregulated following treatment with JQ1 and in *Brd4*^*lacZ/lacZ*^ embryos. (E–G’) Immunohistochemical analysis of pan-STAT3 (E–G’; magenta), with DAPI (E’–G’; nuclei, blue) staining, in mouse blastocysts without inhibitors (control) (E,E’), or treated with 5 μM JQ1 (F,F’) or with 1 μM Stattic (G,G’) for 6 h (Stage 3 to 4). (H–M) Immunohistochemical analysis of phosphorylated (p)STAT3 (H–J’, magenta), with DAPI (H’–J’,blue) staining, and whole-mount *in situ* hybridization of *Nanog* mRNA (K–M) without chemical reagents (control) (H,H’, K), treated with 5× leukemia inhibitory factor (LIF) (I,I’, L) or a combination of 5 μM JQ1 and 5×LIF (J,J’, M) for 6 h of E3.5 blastocysts (Stage 3 to 4). The sample numbers analyzed for each experiment are indicated in Additional file 19. Scale bars: 25 μm in K–M; 40 μm in A–J’. https://doi.org/10.6084/m9.figshare.19134905

**Additional file 7: Fig. S6.** Transcriptome analysis with JQ1 or Stattic treatment in blastocysts (Stage 3 to 4). (A–H) Whole-mount *in situ* hybridization of mouse blastocysts treated without Stattic (A,E,C,G) or with 5 μM Stattic for 3 h (B,F) and 1 μM Stattic for 6 h (D,H) (Stage 3 to 4). Expression of *Arid5b* (A–D), or *Trim43a* (E–H) mRNAs. (I)Schematic illustration of the experimental strategy for RNA sequencing. (J) Venn diagram showing similarities and differences in the RNA-sequence (seq) expression profile of E3.5 blastocysts treated with 5 μM JQ1 or 1 μM Stattic for 6 h. The number of JQ1-downregulated (sensitive) genes was greater than that of Stattic-downregulated (sensitive) genes. The % indicates the ratio of down-regulated genes to all expressed genes. (K) The fraction of genes down-regulated by JQ1 (left) and Stattic (right), respectively. (L) Box-and-whiskers plots of fold changes in Stattic-sensitive genes (*n*=110) versus Stattic-insensitive genes (*n*=1,263) upon treatment with 5 μM JQ1 for 6 h of E3.5 blastocysts. The fold change was determined by RNA-seq. Each box includes values within the 25th and 75th percentiles (with the median highlighted by the middle line), and whiskers extend from the 10th to the 90th percentile (one-tailed Mann–Whitney’s *U*-test, *p* < 0.001). Average fold changes: -2.9119 for Stattic-insensitive, and -4.0615 for Stattic-sensitive genes. The sample numbers analyzed for each experiment are indicated in Additional file 19. Scale bars: 25 μm in A–H. https://doi.org/10.6084/m9.figshare.19134911

**Additional file 8.** RNA-seq data in mouse blastocysts. https://doi.org/10.6084/m9.figshare.19134914

**Additional file 9.** Riken number converted to gene symbol. https://doi.org/10.6084/m9.figshare.19134920

**Additional file 10: Fig. S7.** List of the selected genes specifically down-regulated with Stattic and/or JQ1 treatment (Stages 3 to 4). (A) List of all genes associated with the term “stem cell population maintenance” by Gene Ontology analysis in Stattic-sensitive (overlapped) genes (*n*=110) or Stattic insensitive genes (*n*=1,263) (excluding overlapped genes) among JQ1-sensitive genes. (B) List of the top selected genes specifically down-regulated with Stattic treatment. JQ1 down-regulated genes were excluded from the list with RNA-sequencing. The list shows the top 15 genes that displayed a larger fold change of Stattic-sensitive and JQ1-insensitive genes among 202 genes. https://doi.org/10.6084/m9.figshare.19134926

**Additional file 11: Fig. S8.** The epiblast lineage was not maintained properly in *Brd4*^*lacZ/lacZ*^ blastocysts (Stage 4). (A–D””) Immunohistochemical analysis of NANOG (magenta), GATA6 (green), and CDX2 (yellow), with DAPI (nuclei, blue) staining, in wild type (WT) (A,C–C””) or *Brd4*^*lacZ/lacZ*^ embryos (B,D–D””) at E4.25. (E) The ratio of numbers of NANOG-positive (magenta), GATA6-positive (green), double-positive (DP, white), and double-negative cells (DN, grey) within DAPI-positive inner cell mass (ICM)–derived inner cells of WT or *Brd4*^*lacZ/lacZ*^ embryos at E4.25. DP cells in WT and *Brd4*^*lacZ/lacZ*^ embryos are 0% and 2.5%, respectively. DN cells in WT and *Brd4*^*lacZ/lacZ*^ embryos are 0.9% and 2.6%, respectively. (F) The total number of ICM-derived inner cells in WT or *Brd4*^*lacZ/lacZ*^ embryos at E4.25 (two-tailed Mann–Whitney’s *U*-test, ***p < 0.001). (G) The numbers of NANOG- and GATA6-positive cells in WT or *Brd4*^*lacZ/lacZ*^ ICM-derived inner cells at E4.25 (two-tailed Mann–Whitney’s *U*-test, **p < 0.01). (H,I) Immunohistochemical analysis of phospho-histone H3 (pH3; green), with DAPI (blue) staining, in WT (H) or *Brd4*^*lacZ/lacZ*^ embryos (I) at E4.25. (J) The total number of pH3-expressing cells in WT or *Brd4*^*lacZ/lacZ*^ embryos at E4.25 (two-tailed Mann–Whitney’s *U*-test, *p < 0.05). (K,L) Immunohistochemical analysis of cleaved caspase-3 (green), with DAPI (blue) staining, in WT (K) or *Brd4*^*lacZ/lacZ*^ embryos (L) at E4.25. (M) The total number of caspase-3–expressing cells in WT or *Brd4*^*lacZ/lacZ*^ embryos at E4.25 (two-tailed Mann–Whitney’s *U*-test, *p < 0.05). Red lines indicate the mean value and green lines represent SE bars (F,G,J,M). Individual values of markers-expressing cells are provided in Additional file 15. The sample numbers analyzed for each experiment are indicated in Additional file 19. Scale bars: 25 μm in C–C””,D–D””; 40 μm in H,I,K,L; 50 μm in A,B. https://doi.org/10.6084/m9.figshare.19134947

**Additional file 12: Fig. S9.** The epiblast lineage is unaffected in *Brd2-*deficient blastocysts (Stage 3). (A–E’) Immunohistochemical analysis of BRD2 protein (A–E; magenta), with DAPI (A’ –E’; nuclei, blue) staining, in the wild type (WT) at 8-cell (A,A’), 16-cell (B,B’), E3.5 (C,C’), E4.5 (D,D’), and *Brd2*^*tg/tg*^ embryos at E3.5 (E,E’). Expression of BRD2 protein is undetectable in a *Brd2*^*tg/tg*^ embryo (E). (F,G) Whole-mount *in situ* hybridization analysis of *Nanog* mRNA in WT (F) and *Brd2*^*tg/tg*^ E3.5 blastocysts (G). (H,I) Immunohistochemical analysis of NANOG (magenta), with DAPI (blue) staining, in WT (H) or *Brd2*^*tg/tg*^ blastocysts at E3.5 (I). NANOG expression was not reduced in a *Brd2*^*tg/tg*^ blastocyst (I). (J) The ratio of numbers of NANOG-expressing cells to DAPI-positive cells (nuclei) in WT and *Brd2*^*tg/tg*^ blastocysts (two-tailed Mann–Whitney’s *U*-test, n.s.: not significant; *p* = 0.669). Red lines indicate mean values and green lines represent SE bars. (K) The total number of DAPI-positive cells after electroporation in the absence of sgRNA or Cas9 protein, and without electroporation following 75 hours of culturing of CD-1 zygotes (two-tailed Mann–Whitney’s *U*-test, **p < 0.01). Electroporation can delay normal development. Red lines indicate mean values and green lines represent SE bars. Individual values of markers-expressing cells are provided in Additional file 15. The sample numbers analyzed for each experiment are indicated in Additional file 19. Scale bars: 25 μm in A–B’,F,G; 40 μm in C,C’,E,E’,H,I; 50 μm in D,D’. https://doi.org/10.6084/m9.figshare.19134956

**Additional file 13.** Antibody information. https://doi.org/10.6084/m9.figshare.19136207

**Additional file 14.** Set value information of ratio analysis for cell counting. https://doi.org/10.6084/m9.figshare.19136393

**Additional file 15.** Numbers of markers-expressing cells and DAPI-positive cells in Figs. 2O, 3S, 5D, 5Y, 6D, 7F, S1D, S2G, S4N, S8F, S8G, S8J, S8M, S9J, and S9K. https://doi.org/10.6084/m9.figshare.19136894

**Additional file 16.** Intensity value of pSTAT3 in Additional file 5: Figure S4G. https://doi.org/10.6084/m9.figshare.19136897

**Additional file 17.** PCR primer sets and their amplified lengths used for generatinon of in situ hybridization RNA probes. https://doi.org/10.6084/m9.figshare.19136903

**Additional file 18.** Genomic regions for RNA-FISH probes and their lengths. https://doi.org/10.6084/m9.figshare.19136909

**Additional file 19.** The number of samples for experiments. https://doi.org/10.6084/m9.figshare.19136912

## Data Availability

All data generated or analyzed during this study are included in this published article and the accompanying additional files. RNA-seq data and fastq files were deposited with DNA Data Bank of Japan (DDBJ) (Accession number DRA012031 [https://ddbj.nig.ac.jp/resource/sra-submission/DRA012031], PRJDB11652; Project title: Transcriptomic analysis of mouse preimplantation embryos treated with JQ1 and Stattic submitted by Isao Matsuo).

## Abbreviations

BET: Bromodomain and extra-terminal domain
DAPI: 4′,6-Diamidino-2-phenylindole
ES: Embryonic stem
FGF: Fibroblast growth factor
GO: Gene Ontology
ICM: Inner cell mass
IL: Interleukin
IPA: Ingenuity Pathway Analysis
JQ1: (+)-JQ1
JAK: Janus kinase
LIF: Leukemia inhibitory factor
PrE: Primitive endoderm
pSTAT3: Phosphorylated form of STAT3
P-TEFb: Positive transcription elongation factor b
RNA-seq: RNA sequencing
Sg: Single guide
STAT3: Signal transducer and activator of transcription 3
TE: Trophectoderm
