# Supplementary figures and images for: BET proteins are essential for the specification and maintenance of the epiblast lineage in mouse preimplantation embryos
Source: BMC Biol. 2022 Mar 9;20:64. doi: 10.1186/s12915-022-01251-0 (PMC8905768; doi:10.1186/s12915-022-01251-0)

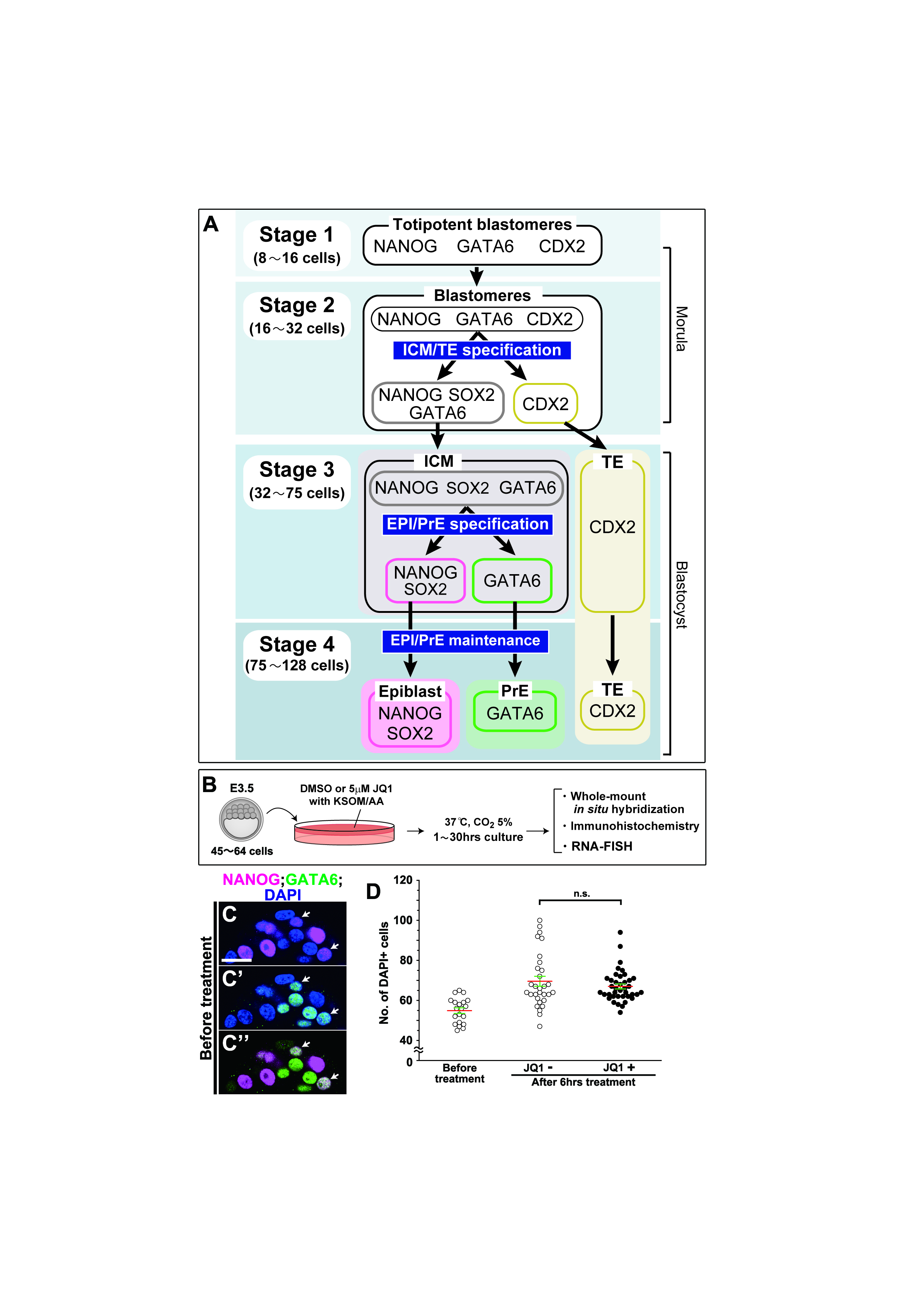

Supplement: Supplementary file 1 — Additional file 1: Fig. S1. Schematic illustrations of our experimental strategy and embryonic stages before and after JQ1 treatment in mouse preimplantation development. (A) Schematic illustration of the formation of three cell lineages and expression of lineage-specific markers during mouse preimplantation development. The number of total cells in embryos are depicted on the left side along with the preimplantation stages as described in Results (“Stage 1” to “Stage 4”). (B) Schematic illustration of the experimental strategy for mouse E3.5 blastocyst (45 to 64 cells) culture without or with JQ1. (C–C”) Immunohistochemical analysis of a 56-cell stage E3.5 blastocyst before treatment (Stage 3). NANOG (magenta), GATA6 (green), and DAPI (nuclei, blue) staining; and a merged view without DAPI staining. E3.5 blastocysts (45 to 64 cells) were composed of mostly co-expressing inner cell mass (ICM; white, arrows) and mutually exclusive, salt and pepper ICM (magenta or green) cells in terms of NANOG and GATA6 (C”). (D) The number of total cells (DAPI-positive cells) before, after without JQ1 or with 5 μM JQ1 6 h-treatment of E3.5 blastocysts (two-tailed Mann–Whitney’s U-test, n.s.: not significant; p = 0.804). Red lines indicate mean values and green lines represent SE bars. Individual values of markers-expressing cells are provided in Additional file 15. The sample numbers analyzed for each experiment are indicated in Additional file 19. Scale bars: 20 μm in C–C”. DMSO, dimethyl sulfoxide; EPI, epiblast; PrE, primitive endoderm; RNA–FISH, RNA–fluorescence in situ hybridization; TE, trophectoderm. https://doi.org/10.6084/m9.figshare.19126595 [file 12915_2022_1251_MOESM1_ESM.jpg]

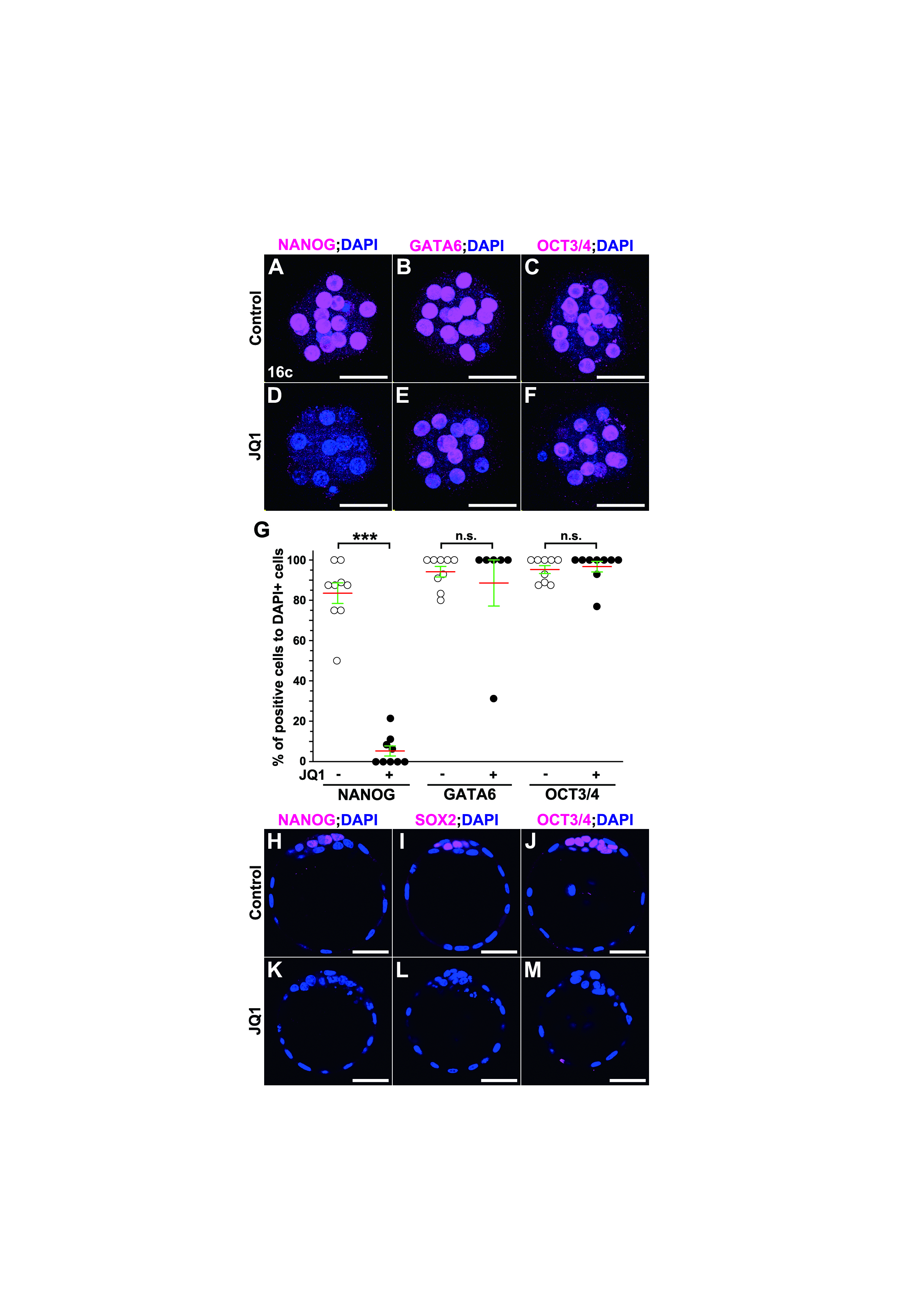

Supplement: Supplementary file 2 — Additional file 2: Fig. S2. Expression of lineage-specific markers after JQ1 treatment of morulae (Stage 1) and blastocysts (Stage 4). (A–F) Immunohistochemical analysis of 16-cell stage morulae (Stage 1) treated without JQ1 (control; A–C) or with 5 μM JQ1 (D–F) for 6 h of 8-cell blastomeres. NANOG (A,D), GATA6 (B,E), and OCT3/4 (C,F) (magenta), with DAPI (nuclei, blue) staining. (G) The ratio of numbers of NANOG-, GATA6-, or OCT3/4-expressing cells to DAPI-positive cells (nuclei) treated without JQ1 or with 5 μM JQ1 for 6 h of E2.5 embryos (Stage 1) (two-tailed Mann–Whitney’s U-test, ***p < 0.001, n.s.: not significant; GATA6; p = 0.483, OCT3/4; p = 0.399). Red lines indicate the mean value and green lines represent SE bars. (H–M) Immunohistochemical analysis of NANOG (H,K), SOX2 (I,L), and OCT3/4 (J,M) (magenta), with DAPI (H–M, blue) staining, in E3.5 blastocysts cultured without JQ1 (control) or with 5 μM JQ1 for 18 h (Stage 3 to 4). Individual values of markers-expressing cells are provided in Additional file 15. The sample numbers analyzed for each experiment are indicated in Additional file 19. Scale bars: 40 μm in A–F, H–M. https://doi.org/10.6084/m9.figshare.19134794 [file 12915_2022_1251_MOESM2_ESM.jpg]

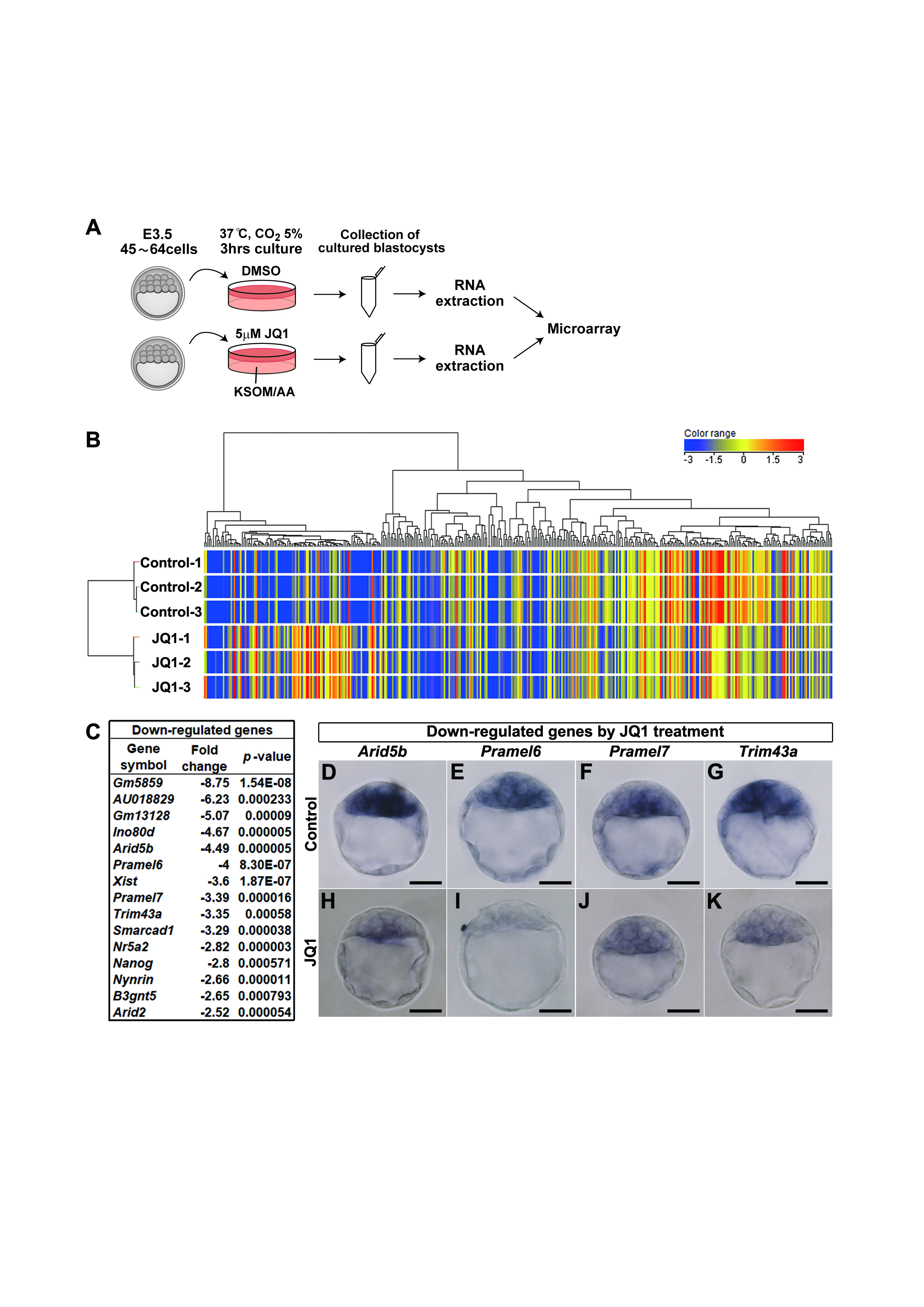

Supplement: Supplementary file 3 — Additional file 3: Fig. S3. Identification of down-regulated genes in JQ1-treated mouse blastocysts with microarray (Stage 3). (A) Schematic illustration of the experimental method for microarray. (B) Heatmap of the hierarchical clustering of down- and up-regulated genes following treatment without JQ1 (control) or with 5 μM JQ1 for 3 h (>2.0-fold, p < 0.05). A red color indicates up-regulated expression, yellow means unchanged expression, and blue indicates down-regulated expression. n=3 independent samples for each experimental condition. Color indicates bi-weight average signal (log2). (C) List of the top 15 down-regulated genes after JQ1 treatment in mouse blastocysts after microarray analysis. The listed genes are selected among genes having a bi-weight average signal (log2) of control blastocysts larger than 8.5. (D–K) Whole-mount in situ hybridization of down-regulated (Arid5b, Pramel6, Pramel7, Trim43a) genes identified by microarray analysis of mouse E3.5 blastocysts treated without JQ1 (D–G) or with 5 μM JQ1 (H–K) for 3 h. The sample numbers analyzed for each experiment are indicated in Additional file 19. Scale bars: 25 μm in D–K. DMSO, dimethyl sulfoxide. https://doi.org/10.6084/m9.figshare.19134857 [file 12915_2022_1251_MOESM3_ESM.jpg]

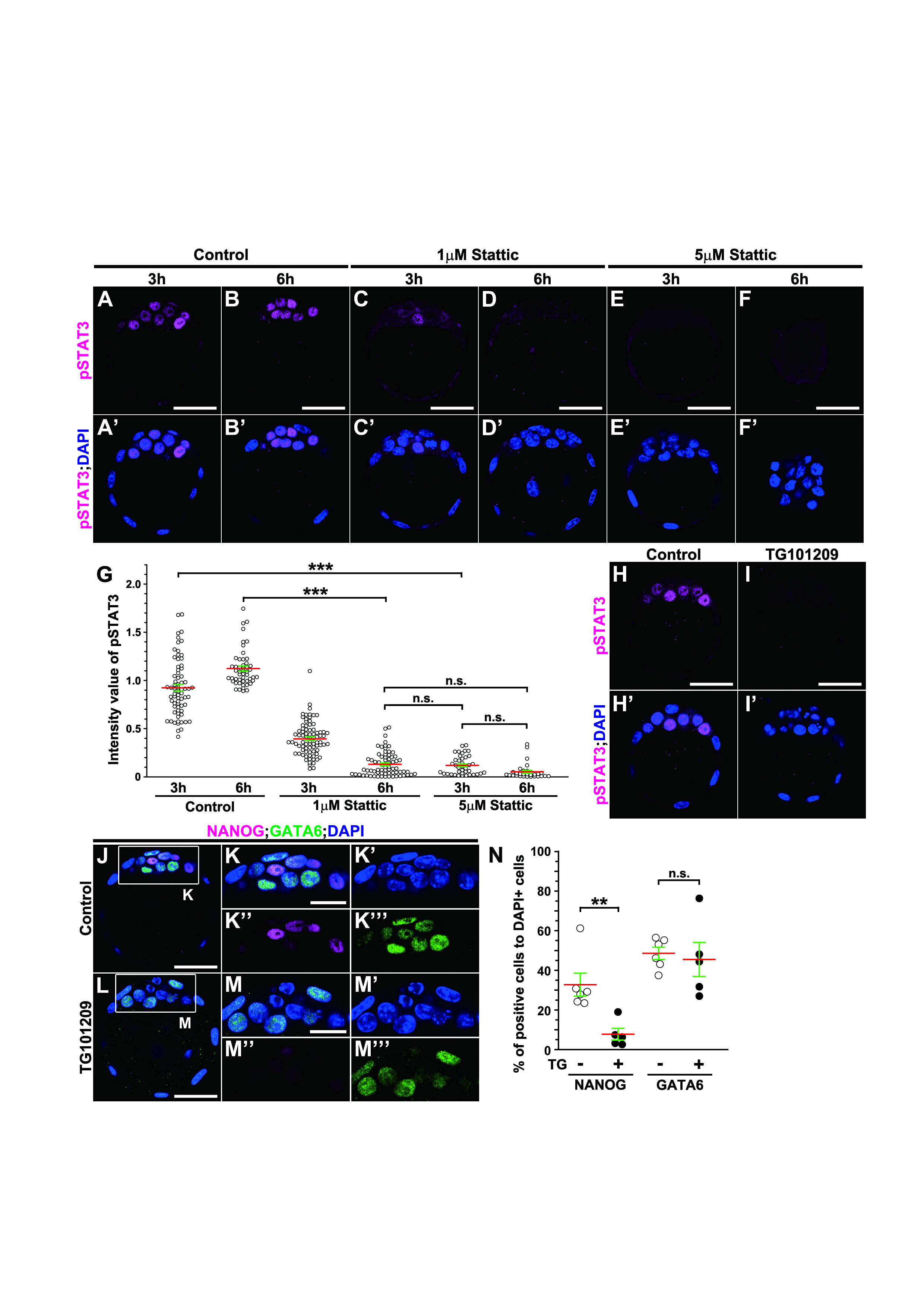

Supplement: Supplementary file 5 — Additional file 5: Fig. S4. Impact of consideration of Stattic concentration on pSTAT3 expression and JAK2 inhibitor on pSTAT3 expression and NANOG-positive cells (Stages 3 and 4). (A–F’) Immunohistochemical analysis of phosphorylated (p)STAT3 (A–F’, magenta), with DAPI (A’–F’, blue) staining, in mouse E3.5 blastocysts without Stattic (control) (A,A’), or treated with 1 μM (C,C’) and 5 μM Stattic (E,E’) for 3 h and without Stattic (control) (B,B’) or treated with 1 μM (D,D’) and 5 μM Stattic (F,F’) for 6 h. (G) Quantification of intensity values of pSTAT3 expression in ICM of E3.5 blastocysts treated without Stattic (control), or treated with 1 μM and 5 μM Stattic for 3h and 6h (one-way ANOVA followed by Tukey-Kramer test; ***p < 0.001, n.s.: not significant; 1 μM Stattic for 6h vs. 5 μM Stattic for 3h, p = 1.000; 1 μM Stattic for 6h vs. 5 μM Stattic for 6h, p = 0.425; 5 μM Stattic for 3h vs. 5 μM Stattic for 6h, p = 0.692). Red lines indicate mean values and green lines represent SE bars. Individual intensity values of pSTAT3 were provided in Additional file 16. (H–I’) Immunohistochemical analysis of pSTAT3 (H–I’, magenta), with DAPI (H’,I’, blue) staining, in mouse E3.5 blastocysts without TG101209 (control; H,H’), or treated with 500 nM TG101209 (I,I’) for 6 h. (J–M”’) Immunohistochemical analysis of NANOG (magenta) and GATA6 (green), with DAPI (nuclei, blue) staining, in mouse blastocysts (Stage 3 to 4) without TG101209 (control; J–K”’), or treated with 500 nM TG101209 (L–M”’) for 6 h. (N) The ratio of numbers of NANOG- or GATA6-expressing cells to DAPI-positive cells (nuclei) treated without TG101209 or 500 nM TG101209 (TG) for 6 h in E3.5 embryos (two-tailed Mann–Whitney’s U-test, **p < 0.01, n.s.: not significant; p = 0.537). Red lines indicate the mean value and green lines represent SE bars. Individual values of markers-expressing cells are provided in Additional file 15. The sample numbers analyzed for each experiment are indicated in Additional file 19. S [file 12915_2022_1251_MOESM5_ESM.jpg]

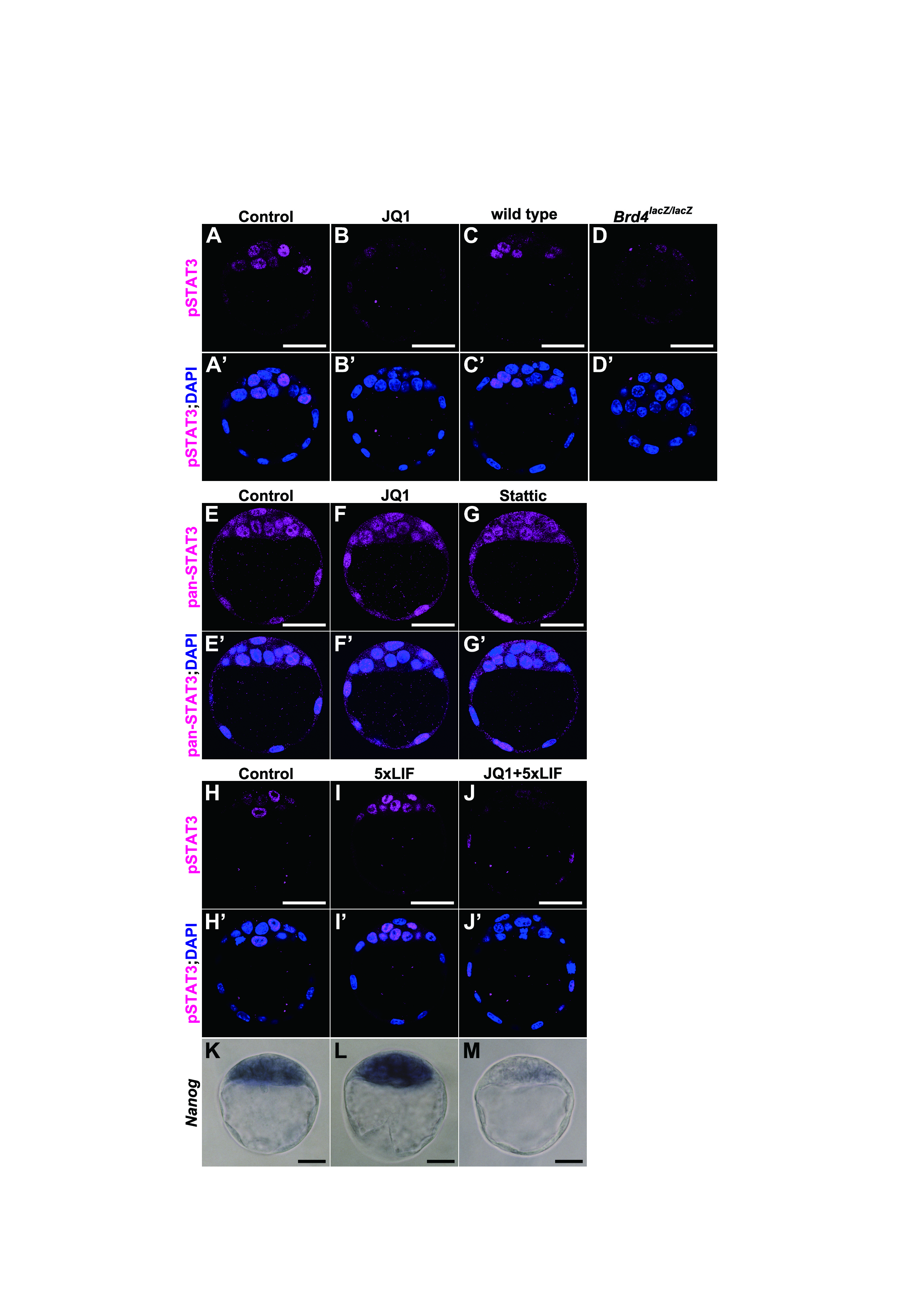

Supplement: Supplementary file 6 — Additional file 6: Fig. S5. Impact of JQ1 treatment, Brd4lacZ/lacZ embryos and additional LIF on JQ1 treatment on pSTAT3 expression (Stage 3 to 4). (A–D’) Immunohistochemical analysis of phosphorylated STAT3 (A–D’, magenta), with DAPI (A’ –D’, blue) staining, in mouse E3.5 blastocysts without JQ1 (control) (A,A’), or treated with 5 μM JQ1 (B,B’) for 6 h; and cultured E3.5 blastocysts of wild type (C,C’) and Brd4lacZ/lacZ embryos (D,D’) for 6 h (Stages 3 and 4). The expression of pSTAT3 shown in here was significantly downregulated following treatment with JQ1 and in Brd4lacZ/lacZ embryos. (E–G’) Immunohistochemical analysis of pan-STAT3 (E–G’; magenta), with DAPI (E’–G’; nuclei, blue) staining, in mouse blastocysts without inhibitors (control) (E,E’), or treated with 5 μM JQ1 (F,F’) or with 1 μM Stattic (G,G’) for 6 h (Stage 3 to 4). (H–M) Immunohistochemical analysis of phosphorylated (p)STAT3 (H–J’, magenta), with DAPI (H’–J’,blue) staining, and whole-mount in situ hybridization of Nanog mRNA (K–M) without chemical reagents (control) (H,H’, K), treated with 5× leukemia inhibitory factor (LIF) (I,I’, L) or a combination of 5 μM JQ1 and 5×LIF (J,J’, M) for 6 h of E3.5 blastocysts (Stage 3 to 4). The sample numbers analyzed for each experiment are indicated in Additional file 19. Scale bars: 25 μm in K–M; 40 μm in A–J’. https://doi.org/10.6084/m9.figshare.19134905 [file 12915_2022_1251_MOESM6_ESM.jpg]

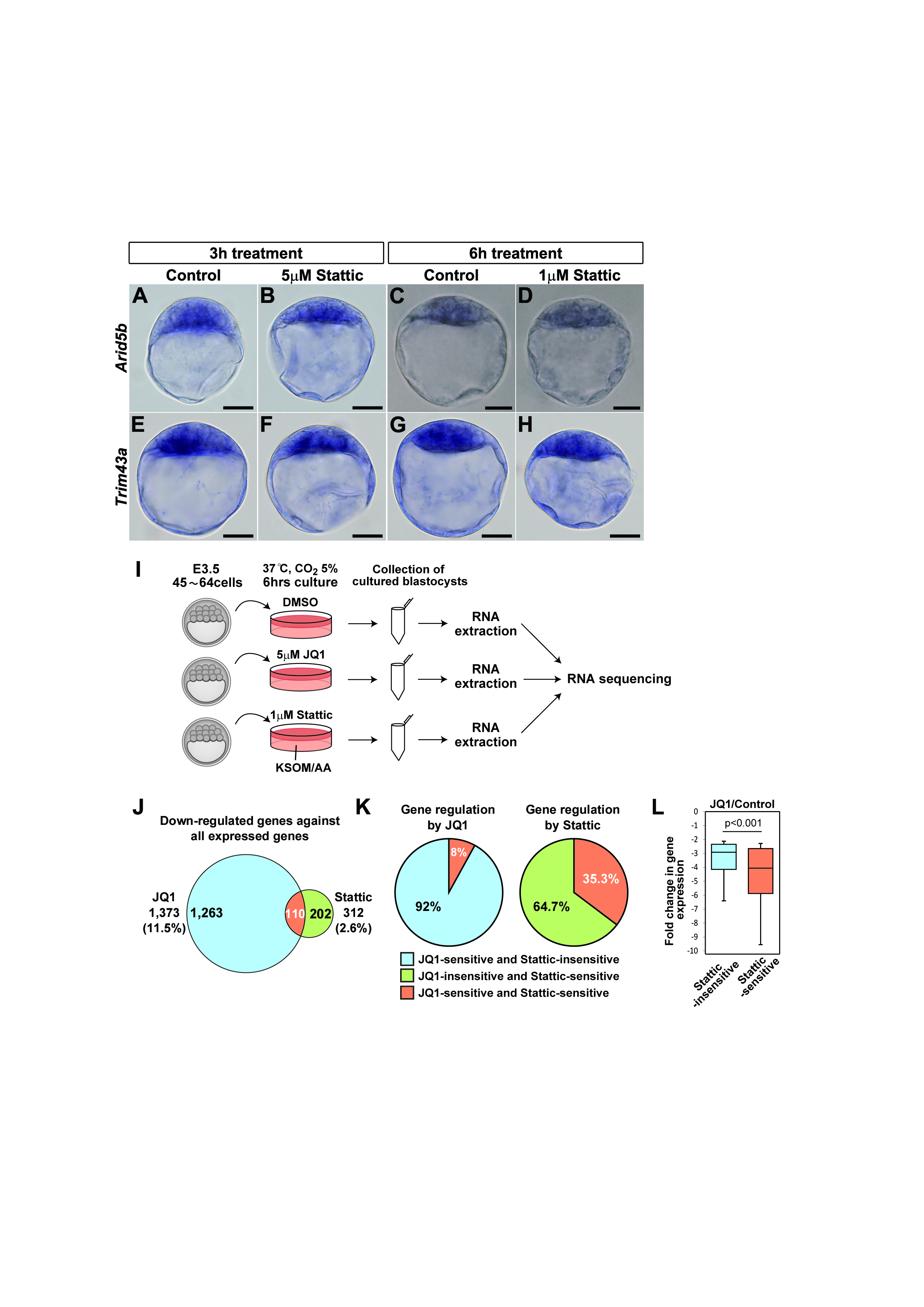

Supplement: Supplementary file 7 — Additional file 7: Fig. S6. Transcriptome analysis with JQ1 or Stattic treatment in blastocysts (Stage 3 to 4). (A–H) Whole-mount in situ hybridization of mouse blastocysts treated without Stattic (A,E,C,G) or with 5 μM Stattic for 3 h (B,F) and 1 μM Stattic for 6 h (D,H) (Stage 3 to 4). Expression of Arid5b (A–D), or Trim43a (E–H) mRNAs. (I)Schematic illustration of the experimental strategy for RNA sequencing. (J) Venn diagram showing similarities and differences in the RNA-sequence (seq) expression profile of E3.5 blastocysts treated with 5 μM JQ1 or 1 μM Stattic for 6 h. The number of JQ1-downregulated (sensitive) genes was greater than that of Stattic-downregulated (sensitive) genes. The % indicates the ratio of down-regulated genes to all expressed genes. (K) The fraction of genes down-regulated by JQ1 (left) and Stattic (right), respectively. (L) Box-and-whiskers plots of fold changes in Stattic-sensitive genes (n=110) versus Stattic-insensitive genes (n=1,263) upon treatment with 5 μM JQ1 for 6 h of E3.5 blastocysts. The fold change was determined by RNA-seq. Each box includes values within the 25th and 75th percentiles (with the median highlighted by the middle line), and whiskers extend from the 10th to the 90th percentile (one-tailed Mann–Whitney’s U-test, p < 0.001). Average fold changes: -2.9119 for Stattic-insensitive, and -4.0615 for Stattic-sensitive genes. The sample numbers analyzed for each experiment are indicated in Additional file 19. Scale bars: 25 μm in A–H. https://doi.org/10.6084/m9.figshare.19134911 [file 12915_2022_1251_MOESM7_ESM.jpg]

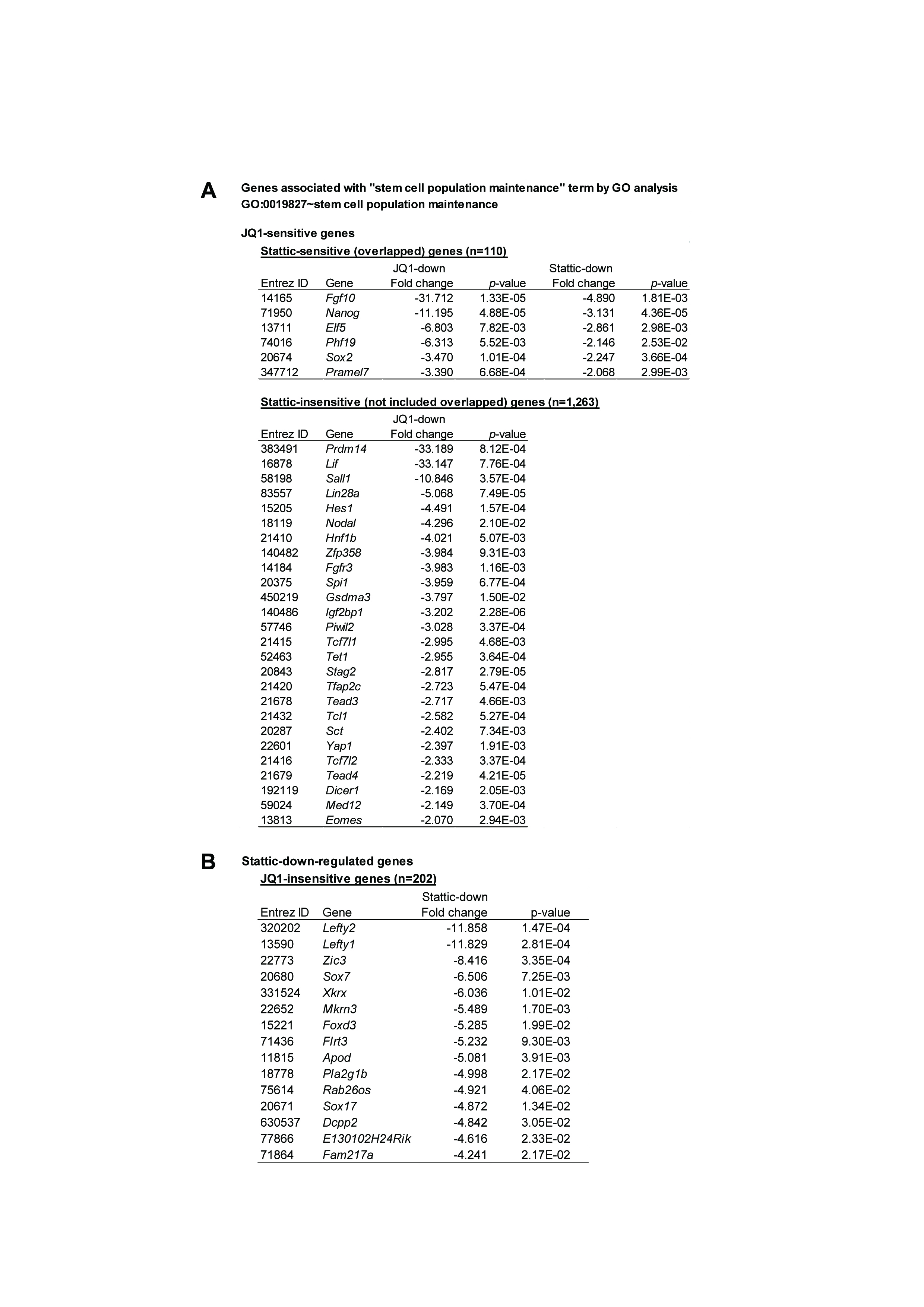

Supplement: Supplementary file 10 — Additional file 10: Fig. S7. List of the selected genes specifically down-regulated with Stattic and/or JQ1 treatment (Stages 3 to 4). (A) List of all genes associated with the term “stem cell population maintenance” by Gene Ontology analysis in Stattic-sensitive (overlapped) genes (n=110) or Stattic insensitive genes (n=1,263) (excluding overlapped genes) among JQ1-sensitive genes. (B) List of the top selected genes specifically down-regulated with Stattic treatment. JQ1 down-regulated genes were excluded from the list with RNA-sequencing. The list shows the top 15 genes that displayed a larger fold change of Stattic-sensitive and JQ1-insensitive genes among 202 genes. https://doi.org/10.6084/m9.figshare.19134926 [file 12915_2022_1251_MOESM10_ESM.jpg]

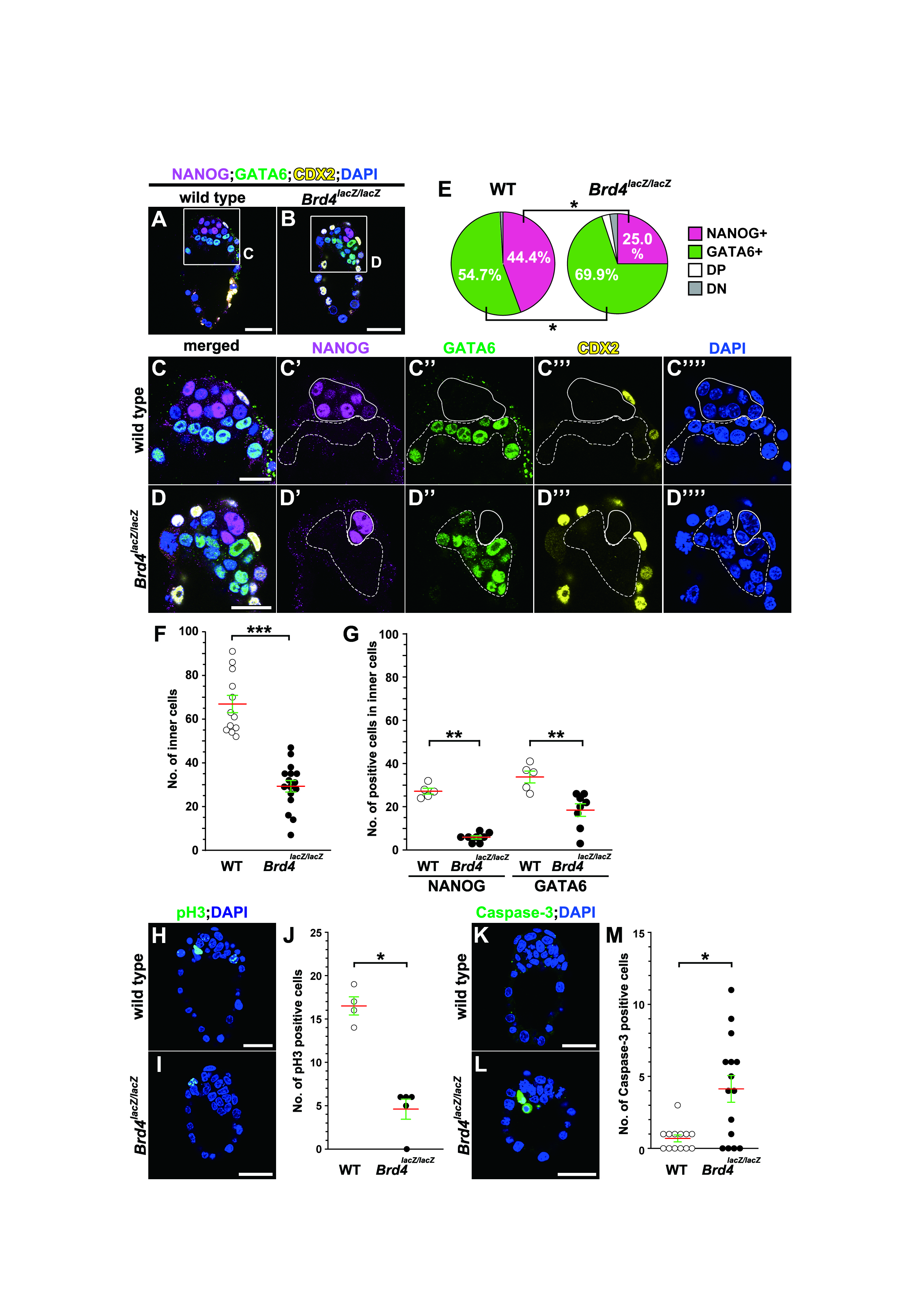

Supplement: Supplementary file 11 — Additional file 11: Fig. S8. The epiblast lineage was not maintained properly in Brd4lacZ/lacZ blastocysts (Stage 4). (A–D””) Immunohistochemical analysis of NANOG (magenta), GATA6 (green), and CDX2 (yellow), with DAPI (nuclei, blue) staining, in wild type (WT) (A,C–C””) or Brd4lacZ/lacZ embryos (B,D–D””) at E4.25. (E) The ratio of numbers of NANOG-positive (magenta), GATA6-positive (green), double-positive (DP, white), and double-negative cells (DN, grey) within DAPI-positive inner cell mass (ICM)–derived inner cells of WT or Brd4lacZ/lacZ embryos at E4.25. DP cells in WT and Brd4lacZ/lacZ embryos are 0% and 2.5%, respectively. DN cells in WT and Brd4lacZ/lacZ embryos are 0.9% and 2.6%, respectively. (F) The total number of ICM-derived inner cells in WT or Brd4lacZ/lacZ embryos at E4.25 (two-tailed Mann–Whitney’s U-test, ***p < 0.001). (G) The numbers of NANOG- and GATA6-positive cells in WT or Brd4lacZ/lacZ ICM-derived inner cells at E4.25 (two-tailed Mann–Whitney’s U-test, **p < 0.01). (H,I) Immunohistochemical analysis of phospho-histone H3 (pH3; green), with DAPI (blue) staining, in WT (H) or Brd4lacZ/lacZ embryos (I) at E4.25. (J) The total number of pH3-expressing cells in WT or Brd4lacZ/lacZ embryos at E4.25 (two-tailed Mann–Whitney’s U-test, *p < 0.05). (K,L) Immunohistochemical analysis of cleaved caspase-3 (green), with DAPI (blue) staining, in WT (K) or Brd4lacZ/lacZ embryos (L) at E4.25. (M) The total number of caspase-3–expressing cells in WT or Brd4lacZ/lacZ embryos at E4.25 (two-tailed Mann–Whitney’s U-test, *p < 0.05). Red lines indicate the mean value and green lines represent SE bars (F,G,J,M). Individual values of markers-expressing cells are provided in Additional file 15. The sample numbers analyzed for each experiment are indicated in Additional file 19. Scale bars: 25 μm in C–C””,D–D””; 40 μm in H,I,K,L; 50 μm in A,B. https://doi.org/10.6084/m9.figshare.19134947 [file 12915_2022_1251_MOESM11_ESM.jpg]

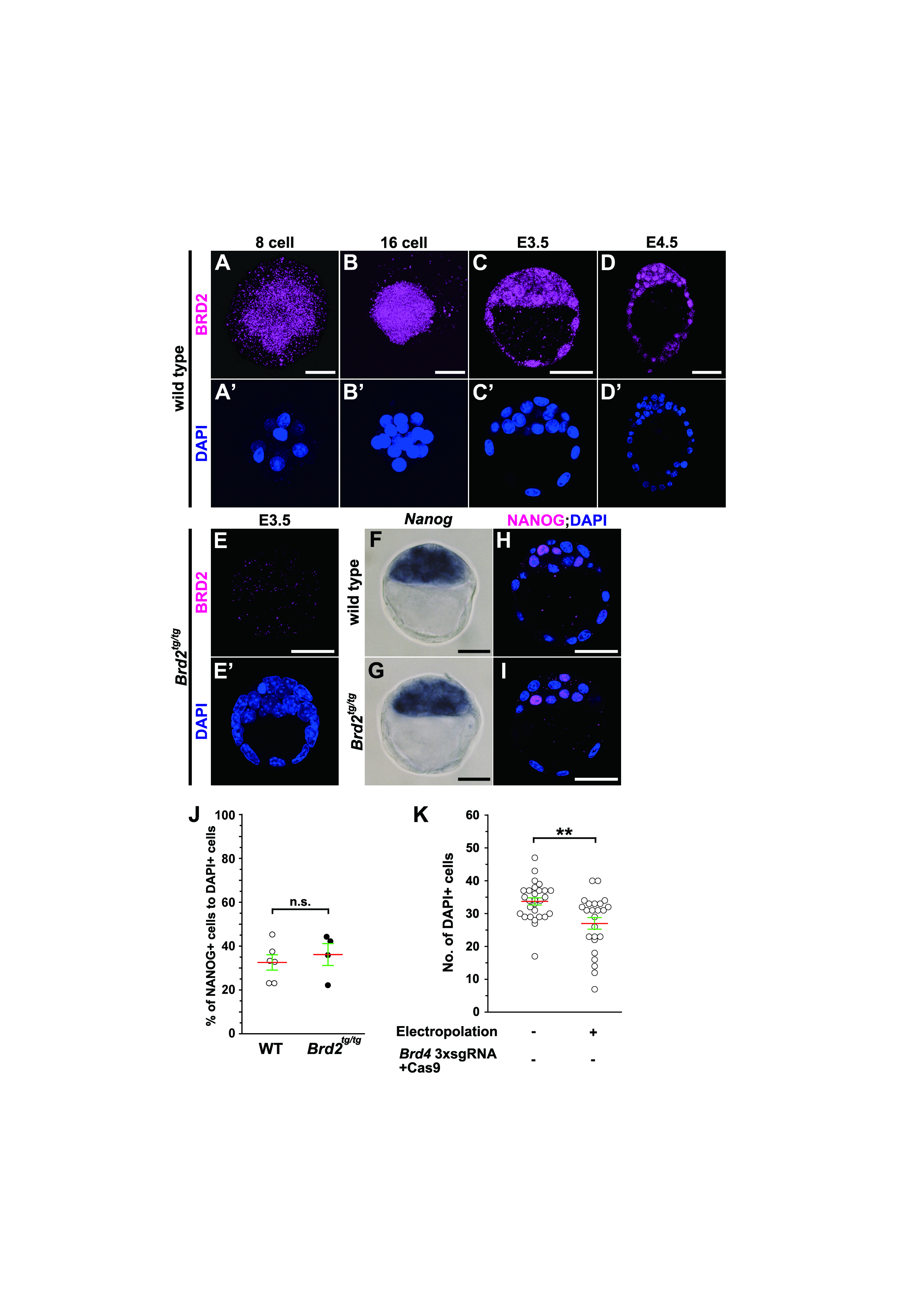

Supplement: Supplementary file 12 — Additional file 12: Fig. S9. The epiblast lineage is unaffected in Brd2-deficient blastocysts (Stage 3). (A–E’) Immunohistochemical analysis of BRD2 protein (A–E; magenta), with DAPI (A’ –E’; nuclei, blue) staining, in the wild type (WT) at 8-cell (A,A’), 16-cell (B,B’), E3.5 (C,C’), E4.5 (D,D’), and Brd2tg/tg embryos at E3.5 (E,E’). Expression of BRD2 protein is undetectable in a Brd2tg/tg embryo (E). (F,G) Whole-mount in situ hybridization analysis of Nanog mRNA in WT (F) and Brd2tg/tg E3.5 blastocysts (G). (H,I) Immunohistochemical analysis of NANOG (magenta), with DAPI (blue) staining, in WT (H) or Brd2tg/tg blastocysts at E3.5 (I). NANOG expression was not reduced in a Brd2tg/tg blastocyst (I). (J) The ratio of numbers of NANOG-expressing cells to DAPI-positive cells (nuclei) in WT and Brd2tg/tg blastocysts (two-tailed Mann–Whitney’s U-test, n.s.: not significant; p = 0.669). Red lines indicate mean values and green lines represent SE bars. (K) The total number of DAPI-positive cells after electroporation in the absence of sgRNA or Cas9 protein, and without electroporation following 75 hours of culturing of CD-1 zygotes (two-tailed Mann–Whitney’s U-test, **p < 0.01). Electroporation can delay normal development. Red lines indicate mean values and green lines represent SE bars. Individual values of markers-expressing cells are provided in Additional file 15. The sample numbers analyzed for each experiment are indicated in Additional file 19. Scale bars: 25 μm in A–B’,F,G; 40 μm in C,C’,E,E’,H,I; 50 μm in D,D’. https://doi.org/10.6084/m9.figshare.19134956 [file 12915_2022_1251_MOESM12_ESM.jpg]
